# The Correlations Between Training Load Parameters and Physical Performance Adaptations in Team Sports: A Systematic Review and Meta-analysis

**DOI:** 10.1186/s40798-025-00952-4

**Published:** 2025-12-11

**Authors:** Filipe Manuel Clemente, Aaron T. Scanlan, Rodrigo Ramirez-Campillo, Diogo Martinho, Rohit Kumar Thapa, Karim Chamari, Rui Miguel Silva, Jason Moran, Hugo Sarmento, Qi Xu, José Afonso

**Affiliations:** 1 Gdansk University of Physical Education and Sport, Gdańsk, 80–336 Poland; 2 Sport Physical Activity and Health Research & Innovation Center, Rio Maior, Portugal; 3 School of Health, Medical and Applied Sciences, Central Queensland University, Rockhampton, Australia; 4 Exercise and Rehabilitation Sciences Institute. School of Physical Therapy. Faculty of Rehabilitation Sciences, Universidad Andres Bello, Santiago, Chile; 5 Research Unit for Sport and Physical Activity (CIDAF), Faculty of Sport Sciences and Physical Education, University of Coimbra, Coimbra, Portugal; 6 Symbiosis School of Sports Sciences, Symbiosis International (Deemed University), Pune, India; 7 Naufar, Wellness and Recovery Center, Doha, Qatar; 8 ISSEP, Higher institute of Sport and Physical Education, ISSEP Ksar Saïd, Manouba University, Tunis, Tunisia; 9 School of Sport, Rehabilitation and Exercise Sciences, University of Essex, Colchester, Essex UK; 10 Centre for Research, Education, Innovation, and Intervention in Sport (CIFI2D), Faculty of Sport, University of Porto (FADEUP), Porto, Portugal

**Keywords:** Heart rate, Locomotor demands, Adaptation, Athletic performance, Aerobic, Neuromuscular, Sprint

## Abstract

**Background:**

Collating evidence on the relationship between training load and physical fitness adaptations in team sport players can help refine training plans.

**Objectives:**

This systematic review and meta-analysis aimed to: (i) identify research designs and methods examining the relationship between training load and physical performance in team sport players, and (ii) synthesize key findings, advancing to meta-analysis where correlations could be established.

**Methods:**

Eligible participants were men and women in team sports at least at the trained or developmental level. Studies included had training loads of at least two weeks with pre-post physical performance evaluations. Searches were conducted in PubMed, Scopus, SPORTDiscus, and Web of Science. Study quality was assessed using the Downs and Black scale, and evidence certainty was evaluated with the GRADE framework.

**Results:**

Of 29,552 records screened, 40 studies with 726 players were included. Significant correlations were evident between heart rate (HR)-based training impulse (TRIMP) and changes in maximal oxygen uptake (*r* = 0.63, *p* < 0.05), velocity at a blood lactate concentration of 2 mmol·L⁻¹ (V_LT_) (*r* = 0.47, *p* < 0.05), and velocity at the onset of blood lactate accumulation (*r* = 0.43, *p* < 0.01). A significant correlation between session-rating of perceived exertion (sRPE) and changes in V_LT_ (*r* = 0.29, *p* < 0.05) was found.

**Conclusions:**

HR-based TRIMP methods to quantify internal training load appear to be effective indicators for potential endurance adaptations around training phases in team sport players. In contrast, sRPE and external training load measures demonstrated limited associations with performance adaptations surrounding training.

**Supplementary Information:**

The online version contains supplementary material available at 10.1186/s40798-025-00952-4.

## Key Points

- Heart rate-based TRIMP largely correlates with endurance adaptations such as VO_2__max_ and lactate thresholds in team sports.
- sRPE shows small correlations with endurance adaptations compared to heart rate-based measures.
- External training load measures exhibit weak correlations with strength, power, and speed, emphasizing the need for further research.

**Supplementary Information:**

The online version contains supplementary material available at 10.1186/s40798-025-00952-4.

## Background

Within team sport environments, a central tenet of training plans is to optimize the physical performance of players through appropriate exercise prescription [1]. Theoretically, training prescription – encompassing exposure to training and competition stimuli [2] – will involve players completing a specific amount of physical work known as the external training load [3]. In turn, the psychophysiological responses to the external training load, referred to as the internal training load, underpin short-term adaptations as well as long-term adaptations with consistent exposure to stimuli over time [4]. The magnitude and frequency of these training stimuli, along with the timing of interventions, can produce positive adaptive or negative maladaptive effects, ultimately influencing physical performance outcomes among players [4].

The relationship between training load (encompassing both training and match stimuli) and subsequent changes in physical performance is often framed within a dose-response paradigm [5]. However, establishing a causal dose-response relationship between these constructs may depend on the chosen framework (e.g., causal exposure-outcome) and methodological approaches adopted (e.g., measure of exposure, metrics) [5]. Moreover, the influence of specific training load measures on physical performance adaptations may vary depending on the sport and competitive level of the players involved. For example, high-speed running volume may be a critical training load outcome to monitor in soccer due to the wide playing area available for counter-attacks and running demands accomplished by players, but this measure may not be useful in basketball [6, 7]. Therefore, it is imperative to understand how correlations between training load and changes in physical performance are influenced by specific demands in team sports.

With several validated and reliable technologies (e.g., global navigation satellite systems [GNSS], inertial measurement units [IMUs]) [8–10] and methods now available to monitor external (e.g., total distance, distances covered at different speed thresholds) and internal training loads (e.g., heart rate, blood lactate) that reflect the physical and psychophysiological demands experienced by team sport players, these advances have also created dilemmas for practitioners regarding which tools and metrics to prioritise, with the proliferation of data encouraging a “more is better” culture that can complicate decision-making [11]. As a result, research interest has increasingly focused on examining the correlations between these load variables and changes in physical performance, as evidenced by two systematic reviews on the topic [12, 13].

More precisely, one review [12] explored the correlations between training load variables and indicators of aerobic, neuromuscular, and game-related statistical performance across various team sports. Some of the studies included in this review exhibited large correlations between high-intensity exercise heart rate (HR) responses and changes in aerobic performance [12]. However, this previous review [12] included studies published in 2018 or earlier, with several studies being published on the topic since. Moreover, it is recommended that many reviews need updating every 2–5 years [14] – meaning a more contemporary synthesis of the literature may be needed in this area. The other review published on this topic [13] explored the correlations between training load variables and changes in aerobic and neuromuscular performance indicators among soccer players aged 14–21 years, showing correlations for various external and internal load variables and performance outcomes [13]. However, this review exclusively examined soccer players who were relatively young, restricting the generalizability of the results to wider age groups and other team sports given the varied demands, training methodologies, and season structures likely to be experienced among them [15]. Additionally, both previous reviews [12, 13] did not meta-analyse findings across studies, meaning subjective interpretations of outcomes collated across studies were reported.

Meta-analysis correlations between training load and changes in physical performance are needed to provide a robust and contemporary understanding of the available evidence, especially considering most original studies on this topic recruited small, context-specific samples that limit wider generalizability. By pooling data, meta-analyses can increase statistical power and reveal patterns or correlations that may not be apparent in individual studies. Furthermore, development of an evidence gap map (EGM) [16] can offer valuable insights on this topic due to the diversity of approaches adopted across studies. Specifically, an EGM can help identify the athlete samples recruited, load and performance outcome variables assessed, study designs employed, and statistical methods applied within the literature to give an overview of the methodological specificities used, pinpoint research gaps, and guide future research directions in the area.

Accordingly, this systematic review with meta-analysis has two primary objectives: (i) identify research designs and methods in studies examining the relationship between training load and physical performance in team sport players; and (ii) synthesize key findings from these studies, advancing to meta-analysis where correlations could be established.

## Methods

Our systematic review addressed the items outlined in the Preferred Reporting Items for Systematic Reviews and Meta-Analyses (PRISMA) 2020 Statement [17].

### Protocol and Registration

The protocol for the systematic review was published on the Open Science Framework (project: osf.io/szu6q; registration: osf.io/z3ysc) on 30 November 2023.

### Eligibility Criteria

The eligibility criteria adhered to the PI/ECOS (Participants, Intervention/Exposure, Comparator, Outcomes, Study Design) framework and are outlined in Table 1. The inclusion criteria encompassed only original research studies published in peer-reviewed journals. There were no restrictions regarding the publication year of studies [18] or the language of the articles.

**Table 1:** Eligibility criteria for this systematic review and meta-analysis

	Inclusion Criteria	Exclusion Criteria
Population	Men or women players engaged in team sports, with the condition that they were not reported as injured or unwell. Players were required to be actively participating in team training and competing at or higher than tier 2 in the Participant Classification Framework* [42], regardless of their age.	Players competing in parasports, or those who have been injured or ill. Furthermore, players competing in individual sports or sporting pursuits not classified as team sports were excluded.
Intervention or/exposure	Observation of training periods lasting at least two weeks where training load (integrating both training sessions and matches) was continuously monitored and quantified between a minimum of two timepoints at which physical performance assessments were undertaken. The two-week period has been established as the shortest duration for observing adaptations in physical performance in team sports [26].	Observations lasting less than two weeks, as well as studies that assess the correlations between training load and physical performance based on a single evaluation timepoint.
Comparator	Studies with and without comparator groups.	Studies in which no team-based training sessions occurred between assessment timepoints, such as during the off-season period.
Outcomes	External (e.g., distance covered, accelerations, and decelerations) and internal training load variables (e.g., heart rate and rating of perceived exertion). Regarding physical performance adaptations, at least two time-points (i.e., baseline and post-exposure) had to be provided for one or more categories including strength, power, speed, endurance, flexibility and/or body composition. In cases where performance was assessed at more than two time-points, consideration of changes between the initial and most recent assessments to be given preference, if feasible.	Studies examining changes in categories not indicative of prominent fitness attributes measured within the performance-based tests such as technical, tactical, psychological, sociological, and well-being variables. Additionally, studies not quantifying training loads between physical performance assessments, or that solely establish correlations between training load and physical performance at a single timepoint, or studies that quantify training load and physical performance variables without analyzing the relationship between them.
Study design	Observational and experimental investigations, including both single-arm and multi-arm approaches.	No restrictions were placed on study design.

* Competitive level was classified based on the Participant Classification Framework [42] where Tier 2 refers to trained/developmental, Tier 3 refers to highly trained/national level, Tier 4 refers to elite/international level, and Tier 5 refers to world class

### Information Sources

Relevant studies were located via searches of PubMed, Scopus, SPORTDiscus, and Web of Science (Core Collection) databases. The initial search was conducted on December 1, 2023, following protocol registration, and an updated search was performed on September 12, 2025. We manually searched the reference lists within included studies to identify further relevant references. Furthermore, we performed snowball citation tracking by utilizing the Web of Science database. To enhance the rigor of the review, insights were also solicited from two external experts of global renown, as verified by Expertscape in the field of team sports (https://expertscape.com/ex/team+sports). Moreover, all studies included in the review underwent thorough examination for potential errata or retractions [44].

### Search Strategy

The search process employed Boolean operators “AND” and “OR”, with a deliberate choice to abstain from employing filters or constraints related to date, language, or study design to optimize the likelihood of identifying relevant studies. The search strategy implemented was as follows:

[Title/Abstract] “team sport*” OR football* OR soccer OR futsal OR handball* OR volleyball* OR basketball* OR hockey OR hurling OR rugby OR cricket OR polo OR lacrosse OR softball OR korfball OR Gaelic* OR netball OR baseball OR “sepak takraw”.

AND.

[Title/Abstract] dose* OR associat* OR correlat* OR relat* OR interaction*.

AND.

[Title/Abstract] load* OR intensit* OR workload* OR volume* OR frequenc* OR duration* OR exposure*.

AND.

[Title/Abstract] physical* OR fitness* OR athletic* OR capacit* OR performance*.

The full search strategy per database can be observed in Supplementary Material 1.

### Selection Process

During the first phase of the search process, the retrieved studies—consisting of titles and abstracts—were independently screened by two authors (FMC and RKT). The abstracts of these identified studies were evaluated against the applicable inclusion criteria. Throughout the second phase of the search process, full-text versions of the retained studies from the first phase were separately screened by the same two authors. Where discrepancies in decisions arose in each phase, the two authors deliberated further and reached consensus in all but three cases, for which additional discussion between them was sufficient, with no need for involvement of a third author (RMS). For effective record management and elimination of duplicates, a combination of manual and automated procedures was employed, facilitated by EndNote™ software (version 20.5, Clarivate Analytics, Philadelphia, PA).

### Data Collection Process

The lead author (FMC) completed the initial phase of data extraction, followed by thorough review for accuracy and comprehensiveness by two further authors (DM and RMS). To facilitate this process, a dedicated Microsoft Excel spreadsheet (Microsoft^®^, USA) was developed to encompass all relevant data. An illustrative excerpt from this datasheet can be found in Supplementary Material 2. In instances where data were missing from the full-text versions of studies, the lead author (FMC) and co-author (DM) contacted the corresponding authors via email and ResearchGate to obtain the necessary information; this occurred on two occasions. If no response was received within three weeks, the data from those studies were excluded from the review and meta-analysis, which was the case for two studies [20, 21].

#### General Information

General details extracted from each included study were the: (i) sample size; (ii) sport, age, sex, and competitive level as defined by the Participant Classification Framework [42] of the players investigated; (iii) seasonal phase during which players were assessed and monitored (defined according to information provided in the studies and generally categorized as pre-season, first half of the season, or second half of the season); and (iv) training frequency and volume (calculated as the product of training frequency and duration) completed by the players. We also collected information on the objectives, design, randomization process, and sampling strategy of each study, along with contextual details such as the time of day each testing session was conducted, the number of rest days prior to each session, the type of familiarization process used for assessment, and whether blinding procedures were employed.

#### Exposure-related Information

To describe the exposure experienced between timepoints at which physical performance was assessed, details extracted included: (i) the number of weeks of training and competition over which loads were monitored; (ii) the number of training sessions and/or matches completed; (iii) training volume (including both training sessions and matches, represented either as a weekly mean or the total sum over the study period, depending on the measures provided in studies); and (iv) the type of training (e.g., physical, technical, tactical) as specified within each study.

### Outcomes

The first outcome is training load, encompassing both external and internal load variables. Training load variables, for training and match scenarios, were extracted when they were accumulated between at least two timepoints at which physical performance was assessed or alternatively as the average weekly load if reported in this manner. Internal load consists of HR-derived measures, which are metrics obtained directly from HR data (e.g., training impulse [TRIMP], time spent in specific HR zones), and rating of perceived exertion (RPE)-derived measures, which involve the use of subjective effort scales (e.g., session-RPE). All measures and their calculation methods were included, specifically accounting for variations in formulas used in some cases, such as TRIMP (e.g., Banister, Edwards) [23], as well as differences in scales applied to RPE (e.g., Borg, Foster) [24]. External load measures include distance-related metrics, which capture total distances accumulated overall and at various speed thresholds (e.g., total distance covered, distance covered at high-speed running), accelerometer-based metrics (e.g., accelerations and decelerations, distance covered during accelerations or decelerations, and composite measures such as Player Load) [25], and session-related metrics, which reflect the characteristics of the training or match sessions (e.g., total training duration).

The second outcome pertains to physical performance adaptations [12], which focuses on differences in physical performance variables between a minimum of two timepoints (i.e., baseline and post-intervention). For the purposes of this review, a minimum duration of two weeks had to be applied between timepoints given previous research has indicated short-term changes in physical performance can manifest within this timeframe [26].

The categories of physical performance were: (i) strength – encompassing maximal strength, the highest amount of force generated in a single effort (e.g., one-repetition maximum in various exercises), and muscular endurance, the ability of a muscle or group of muscles to sustain repeated contractions over time (e.g., the maximum number of repetitions in a single exercise); (ii) power – involving explosive power, which refers to the capacity to generate maximum force in a short period (e.g., standing throwing velocity, jumping height in vertical jump tests), and anaerobic power, the ability to perform high-intensity exercise for brief durations without predominant reliance on oxidative metabolic pathways (e.g., best time in repeated-sprint ability tests); (iii) speed – covering linear speed, the maximum velocity reached when running in a straight or curved path (e.g., best time in linear speed tests over various distances, maximum sprint speed), as well as change-of-direction speed, which is the ability to quickly change movement direction (e.g., best performance in change-of-direction tests); (iv) endurance – including aerobic endurance, the capacity to sustain prolonged physical activity (e.g., maximal oxygen uptake, total distance covered in tests, maximal aerobic speed), and lactate threshold measures, the point during exercise at which a given lactate concentration or accumulation is evident in the blood (e.g., performance indicators taken at certain lactate concentrations). In this category, we deliberately included both physiological determinants (e.g., VO₂_max_, lactate threshold) and performance outcomes (e.g., total distance covered in tests) to capture not only the underlying aerobic capacity but also its translation into functional performance. For instance, Dal Pupo et al. [27] found that physiological measures such as VO₂_max_, velocity at VO₂_max_, and maximal accumulated oxygen deficit, together with neuromuscular performance measures like countermovement jump (CMJ) and SJ (squat jump), were significantly associated with sprint performance. (v) flexibility – consisting of static flexibility, the range of motion around a joint when at rest, and dynamic flexibility, the ability to move a joint through its full range of motion during activity; and (vi) body composition – referring to the proportion of body fat, lean mass or lean muscle mass.

### Risk of Bias Assessment

Quality evaluation of the included studies was conducted by two authors (RMS and FMC), who independently assessed the risk of bias. If agreement could not be reached, a third author (DM) was to be consulted to provide a consensus decision; however, this was not required. Included studies were evaluated for quality using a modified version of the Downs and Black assessment scale [28], as used by Sarmento et al. [29]. Consistent with a previous review [30], the quality scores were categorized as follows: (1) low methodological quality for scores of 50% or lower; (2) good methodological quality for scores ranging from 51% to 75%; and (3) excellent methodological quality for scores exceeding 75%. Inter-rater reliability analysis was performed using Cohen’s kappa value, with excellent agreement observed between authors (κ = 0.94).

### Summary measures, Synthesis of results, and Risk of Publication Bias

Meta-analyses were conducted when at least three independent studies provided effect size measurements for the same physical performance category (irrespective of the test used or variable reported) [31]. Effect sizes were determined using correlation coefficients (r), along with their respective standard errors or sample sizes. In cases where studies utilized multivariate linear regression, we applied a series of transformations to convert unstandardized regression coefficients (β) into r for meta-analysis, as done previously [32, 33].

To account for inherent inaccuracies in estimating between-study variances [34], we employed random-effects models to generate pooled correlation coefficients (r) alongside corresponding 95% confidence intervals (CI) and prediction intervals. The resulting pooled effect sizes for correlation coefficients (r) were categorized as trivial (≤ 0.1), small (0.1 < *r* ≤ 0.3), medium (0.3 < *r* ≤ 0.5), large (0.5 < *r* ≤ 0.7), very large (0.7 < *r* ≤ 0.9), nearly perfect (0.9 < *r* < 1.0), or perfect (1.0) following established guidelines [35]. All statistical analyses were conducted using the Comprehensive Meta-Analysis software (version 2; Biostat, Englewood, NJ, USA).

The extent of heterogeneity across included studies, as determined by Cochran’s Q-statistic [36], was employed to compute I^2^ values which were categorized as follows: low heterogeneity (< 25%), moderate heterogeneity (25–75%), and high heterogeneity (>75%) [36, 37]. Sensitivity analyses were performed to assess the stability of the summary estimates and identify whether any study significantly contributed to the observed heterogeneity. Consequently, each study was systematically removed from the model to evaluate its impact on the overall results.

Risk of publication bias across included studies was evaluated by the expanded version of Egger’s test [38]. Additionally, potential contributors to heterogeneity were investigated including the sport, sex of players investigated, competitive level at which the investigated players competed, study duration, type of load monitored (external and internal), and type of physical performance assessed (strength, power, speed, endurance, flexibility, body composition), by segregating the meta-analyses based on each of these variables [39].

### Certainty Assessment

The evaluation was centered on the five dimensions stipulated in the GRADE [40, 41] including risk of bias, indirectness, inconsistency, risk of publication bias, and imprecision. These dimensions are used to assign a classification of high, moderate, low, or very low quality to the body of evidence pertaining to load and performance outcomes. All studies were non-randomized, commencing at low quality, with possible upgrades contingent upon the presence of substantial effect sizes, adept control of credible confounding variables, and confirmation of a dose-response pattern. However, these upgrades were only implemented in cases where there were no reasons to downgrade, as per GRADE guidelines [42–45].

To assess the certainty of evidence in analyses, we established a set of criteria. First, we scrutinized the risk of bias in the included studies, and if we identified moderate risk in the bias assessment (on average, Downs and Black score below 85%), we downgraded the evidence by one level. In cases where a high risk of bias was apparent (on average, PEDro score below 70%), a more substantial downgrade of two levels was applied. Indirectness in the evidence was considered low by default, as the populations, exposures, and outcomes were considered direct as per the eligibility criteria. Thirdly, we opted not to evaluate the risk of publication bias as the minimum of 10 studies for each analysis was not achieved [38, 39]. Fourthly, when examining inconsistency, if the impact of heterogeneity was moderate (*I*^2^ = 25–75%) [39], we downgraded the evidence by one level, and if the impact of heterogeneity was high (I^2^ >75%), we downgraded the evidence by two levels. Finally, we assessed imprecision by considering the number of players (fewer than 800 players [400 per group] resulted in evidence being downgraded by one level [46]) and the clarity of the effects (no clear direction led to evidence being downgraded by one level – i.e., the 95% CI crosses zero) [46].

## Results

### Study Identification and Selection

**Fig. 1:**
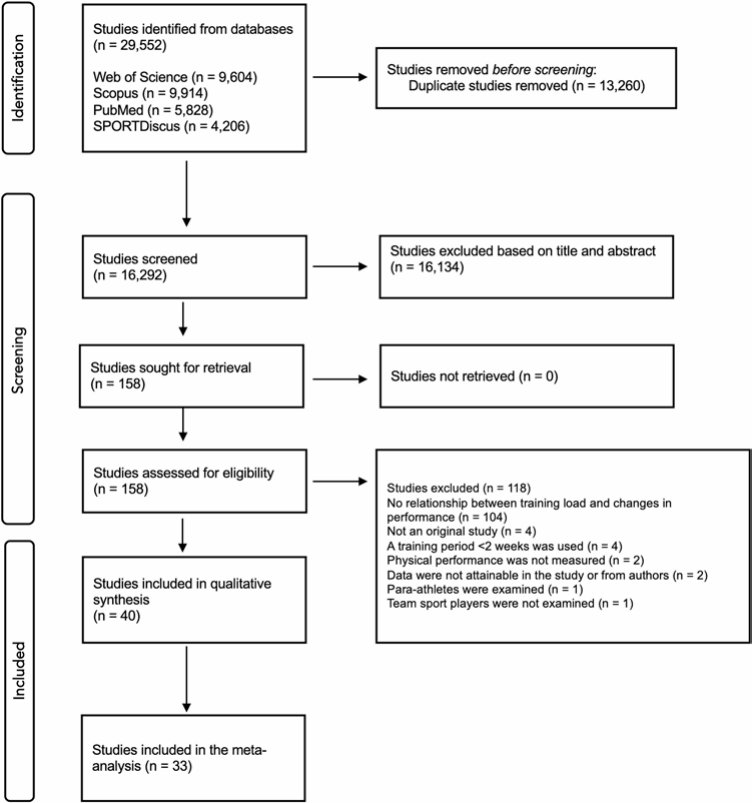
PRISMA flowchart showing the search and screening outcomes for this systematic review and meta-analysis

Database searches collectively identified 29,552 records. Subsequently, duplicates (*n* = 13,260) were removed, and 16,292 studies were screened according to the title and abstract. Based on title and abstract, 16,134 studies were excluded, leaving 158 studies for full-text review. During the full-text examination, 118 studies did not meet the eligibility criteria (see Fig. 1 for detailed explanations). Ultimately, 40 studies were included in the review.

### Risk of Bias Assessment

The assessment of methodological quality for included studies (Table 2) revealed mostly high-quality research. More precisely, 23 studies (59%) achieved an excellent quality rating (> 75%), while the remaining 17 studies (41%) achieved a good quality rating (50–75%).

**Table 2:** Quality assessment of the studies using a modified downs and black checklist [29]

Study	Criteria																Score %
	1	2	3	4	5	6	7	8	9	10	11	12	13	14	15	16	
Gorostiaga et al. [54]	1	1	1	1	0	1	1	0	1	1	1	1	0	1	1	1	81.25%
Stagno et al. [58]	1	1	1	1	0	1	1	0	1	1	1	1	0	1	1	1	81.25%
Granados et al. [55]	1	1	1	1	0	1	1	1	1	1	1	1	0	1	1	0	75.00%
Castagna et al. [79]	1	1	1	1	0	1	1	1	1	1	1	1	0	1	1	1	81.24%
Akubat et al. [93]	1	1	1	1	0	1	1	1	1	1	1	1	1	1	1	1	87.25%
Castagna et al. [94]	1	1	1	1	0	1	1	1	1	1	1	1	0	1	1	1	81.25%
Manzi et al. [74]	1	1	1	1	0	1	1	1	1	1	1	1	0	1	1	1	81.25%
Arcos et al. [95]	1	1	1	1	0	1	1	1	1	1	1	1	0	1	1	0	75.00%
Arcos et al. [92]	1	1	1	1	0	1	1	1	1	1	1	1	0	1	1	0	75.00%
Gil-Rey et al. [60]	1	1	1	1	0	1	1	1	1	1	1	1	1	1	1	0	81.25%
Nakamura et al. [59]	1	1	1	1	0	1	1	1	1	0	1	1	1	1	1	1	81.25%
Malone et al. [48]	1	1	1	1	0	1	1	1	1	1	1	1	1	1	1	1	87.25%
Arcos et al. [89]	1	1	1	1	0	1	1	1	1	1	1	1	0	1	0	0	68.75%
Campos-Vazquez et al. [96]	1	1	1	1	0	1	1	1	1	1	1	1	0	1	1	0	75.00%
Dubois et al. [52]	1	1	1	1	0	1	1	1	1	1	1	1	0	1	1	0	75.00%
Dobbin et al. [50]	1	1	1	1	0	1	1	1	1	0	1	1	1	1	1	1	81.25%
Ferioli et al. [56]	1	1	1	1	0	1	1	1	1	0	1	1	1	1	1	1	81.25%
Fitzpatrick et al. [82]	1	1	1	1	0	1	1	1	1	0	1	1	0	1	1	1	75.00%
Figueiredo et al. [61]	1	1	1	1	0	1	1	1	1	1	1	1	1	1	1	0	81.25%
Taylor et al. [53]	1	1	1	1	0	1	1	1	1	1	1	1	1	1	1	1	87.5%
Clemente et al. [81]	1	1	1	1	0	1	1	1	1	1	1	1	0	1	1	1	81.25%
Clemente et al. [86]	1	1	1	1	0	1	1	1	1	1	1	1	0	1	1	1	81.25%
Daniels et al. [51]	1	1	1	1	0	1	1	1	1	1	1	1	0	1	1	0	75.00%
Rabbani et al. [97]	1	1	1	1	0	1	1	1	1	0	1	1	0	1	1	1	75.00%
Saidi et al. [98]	1	1	1	1	0	1	1	1	1	1	1	1	1	1	1	1	87.5%
Azcárate et al. [99]	1	1	1	1	0	0	1	1	1	1	1	1	0	0	1	1	62.5%
Malone et al. [47]	1	1	1	1	0	1	1	1	1	1	1	1	0	1	1	1	81.25%
Malone et al. [49]	1	1	1	1	0	1	1	1	1	1	1	1	0	0	1	0	68.75%
Papadakis et al. [75]	1	1	1	1	0	1	1	1	1	0	0	1	1	1	1	1	68.75%
Ellis et al. [73]	1	1	1	1	0	1	1	1	1	0	1	1	1	1	1	1	81.25%
Figueiredo et al. [71]	1	1	1	1	0	1	1	1	1	1	1	0	1	0	1	1	75.00%
Kalapotharakos et al. [80]	1	1	1	1	0	1	1	1	1	1	1	0	0	1	0	1	68.75%
Younesi et al. [100]	1	1	1	1	0	1	1	1	1	1	1	0	0	1	1	1	75.00%
Ellis et al. [101]	1	1	1	1	0	1	1	1	1	0	1	0	0	1	1	1	68.75%
Rabbani et al. [102]	1	1	1	1	0	1	1	1	1	0	1	1	1	1	1	1	81.25%
Xiong et al. [103]	1	1	1	1	0	1	1	1	1	1	1	1	0	1	1	1	81.25%
Quan et al. [104]	1	1	1	1	0	1	1	1	1	1	1	1	0	1	1	0	75.00%
Perrotta et al. [105]	1	1	1	1	0	1	1	1	1	1	1	1	1	1	1	0	81.25%
Savolainen et al. [106]	1	1	1	1	0	1	1	1	1	1	1	1	0	1	1	1	81.25%
Phillip et al. [57]	1	1	1	1	0	1	1	1	1	0	1	1	0	1	1	1	81.25%

### Study Characteristics

**Fig. 2:**
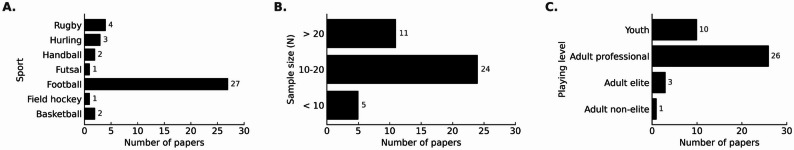
Number of papers published by sport (A), sample size (B) and according to the playing level (C)

The sample characteristics (including sport, sample size, competitive level, sex, age, height, and body mass) are shown in Table 3. Figure 2 (panel A) shows most included studies investigated soccer players (*n* = 27). In turn, multiple studies also examined hurling (*n* = 3) [47–49], rugby league (*n* = 2) [50, 51], rugby union (*n* = 2) [52, 53], handball (*n* = 2) [54, 55] and basketball [56, 57] players, with single studies each examining field hockey [58] and futsal [59] players.

**Table 3:** Characteristics of the players examined in each included study

Study	Sport	Sample size (*N*)	Competitive level	Sex	Age (years)	Stature (m)	Body mass (kg)
Gorostiaga et al. [54]	Handball	15	Tier 5	Men	31.0 ± 7.0	1.88 ± 0.07	95.6 ± 14.3
Stagno et al. [58]	Field hockey	8	Tier 4	Men	24.0 ± 4.0	1.81 ± 0.04	80.8 ± 5.2
Granados et al. [55]	Handball	16	Tier 4	Women	23.1 ± 4.0	1.75 ± 0.06	69.6 ± 8.4
Castagna et al. [79]	Soccer	14	Tier 4	Men	25.0 ± 4.0	1.78 ± 0.07	74.0 ± 8.0
Akubat et al. [93]	Soccer	9	Tier 3	Men	17.0 ± 1.0	1.81 ± 0.01	72.0 ± 6.7
Castagna et al. [94]	Soccer	18	Tier 4	Men	28.6 ± 3.2	1.83 ± 0.06	80.0 ± 5.4
Manzi et al. [74]	Soccer	18	Tier 4	Men	28.4 ± 3.2	1.82 ± 0.05	79.9 ± 5.5
Arcos et al. [95]	Soccer	21	Tier 3	Men	21.0 ± 1.7	1.81 ± 0.06	76.1 ± 7.7
Arcos et al. [92]	Soccer	14	Tier 3	Men	20.6 ± 1.7	1.79 ± 0.06	73.5 ± 7.0
Gil-Rey et al. [60]	Soccer	14	Tier 3	Men	17.6 ± 0.6	1.80 ± 0.06	70.3 ± 4.4
		14	Tier 2	Men	17.5 ± 0.5	1.78 ± 0.06	71.1 ± 6.5
Nakamura et al. [59]	Futsal	10	Tier 3	Men	19.1 ± 0.8	1.75 ± 0.06	71.3 ± 6.6
Malone et al. [48]	Hurling	20	Tier 4	Men	25.5 ± 3.2	1.79 ± 0.03	78.5 ± 4.5
Arcos et al. [89]	Soccer	14	Tier 3	Men	20.6 ± 1.5	1.80 ± 0.01	73.6 ± 7.4
Campos-Vazquez et al. [96]	Soccer	12	Tier 3	Men	20.7 ± 4.3	1.77 ± 0.06	73.1 ± 5.2
Dubois et al. [52]	Rugby union	8	Tier 4	Men	25.8 ± 4.2	NR	88.4 ± 3.1
Dobbin et al. [50]	Rugby league	16	Tier 3	Men	17.2 ± 0.7	1.80 ± 0.05	88.5 ± 10.1
Ferioli et al. [56]	Basketball	18	Tier 4	Men	25.6 ± 6.0	1.98 ± 0.01	95.5 ± 13.0
			Tier 3	Men	23.3 ± 4.7	1.90 ± 0.09	82.2 ± 11.6
Fitzpatrick et al. [82]	Soccer	14	Tier 3	Men	17.1 ± 0.5	1.78 ± 0.05	70.9 ± 5.8
Figueiredo et al. [61]	Soccer	9	Tier 2	Men	14.3 ± 8.3	1.69 ± 0.10	58.8 ± 8.3
		8	Tier 2	Men	16.0 ± 0.7	1.78 ± 0.10	70.5 ± 10.2
Taylor et al. [53]	Rugby union	10	Tier 3	Men	18.4 ± 1.0	1.83 ± 0.06	85.9 ± 13.0
Clemente et al. [81]	Soccer	14	Tier 3	Men	24.9 ± 3.5	1.68 ± 0.04	71.6 ± 8.7
Clemente et al. [86]	Soccer	23	Tier 3	Men	24.7 ± 2.8	1.79 ± 0.06	76.5 ± 5.6
Daniels et al. [51]	Rugby league	21	Tier 4	Men	23.3 ± 4.4	1.81 ± 0.07	91.6 ± 8.9
Rabbani et al. [97]	Soccer	11	Tier 4	Men	27.2 ± 4.5	1.80 ± 0.09	72.7 ± 6.6
Saidi et al. [98]	Soccer	18	Tier 4	Men	20.1 ± 10.4	1.78 ± 0.04	72.6 ± 6.1
Azcárate et al. [99]	Soccer	20	Tier 3	Men	27.1 ± 3.1	1.82 ± 0.05	76.5 ± 5.8
Malone et al. [47]	Hurling	30	Tier 4	Men	26.5 ± 3.1	1.78 ± 0.03	81.5 ± 4.5
Malone et al. [49]	Hurling	30	Tier 4	Men	24.0 ± 4.0	1.80 ± 0.02	78.0 ± 3.0
Papadakis et al. [75]	Soccer	16	Tier 3	Men	25.6 ± 3.2	1.80 ± 0.06	73.3 ± 6.6
Ellis et al. [73]	Soccer	9	Tier 3	Men	17.0 ± 1.0	1.79 ± 0.06	71.3 ± 5.8
Figueiredo et al. [71]	Soccer	16	Tier 3	Men	18.8 ± 1.0	1.75 ± 0.06	68.7 ± 6.5
Kalapotharakos et al. [80]	Soccer	16	Tier 4	Men	26.8 ± 3.8	1.79 ± 0.06	77.8 ± 7.7
Younesi et al. [100]	Soccer	22	Tier 4	Men	27.2 ± 3.4	1.74 ± 0.04	69.1 ± 6.4
Ellis et al. [101]	Soccer	12	Tier 3	Men	17.0 ± 1.0	1.78 ± 0.06	72.1 ± 5.6
Rabbani et al. [102]	Soccer	16	Tier 4	Men	26.4 ± 3.8	1.79 ± 0.06	74.2 ± 3.4
Xiong et al. [103]	Soccer	41	Tier 2	Men	16.4 ± 0.5	1.72 ± 0.04	59.4 ± 2.9
Quan et al. [104]	Soccer	21	Tier 2	Men	17.7 ± 1.7	1.71 ± 0.05	61.8 ± 4.7
Perrotta et al. [105]	Soccer	27	Tier 3	Women	20.6 ± 1.2	1.66 ± 0.07	57.3 ± 6.6
Savolainen et al. [106]	Soccer	35	Tier 3	Women	21.1 ± 2.8	1.66 ± 0.05	64.0 ± 5.0
Phillip et al. [57]	Basketball	12	Tier 3	Women	20.9 ± 1.2	1.85 ± 8.9	86.6 ± 1.2

NR (not reported). Data are presented as mean ± standard deviation for age, stature, and body mass. Competitive level classified as: tier 2: trained/developmental; tier 3: highly trained/national level; tier 4: elite/international level; tier 5: world class

The pooled number of players across all included studies was 726. Five studies recruited a sample < 10 players while 24 studies recruited a sample between 10 and 20 players, and eleven studies recruited a sample >20 players (Fig. 2, panel B). Three studies [56, 60, 61] included more than one competitive level. Four studies recruited women players exclusively, while the remaining studies recruited male players. No studies combined men and women athletes.

Table 4 summarizes the methodological approaches (period of the season examined, weeks of training exposure, training load variables, and physical performance measures) extracted from each study. Regarding season period, 15 studies were conducted during the pre-season phase, 13 studies were performed during the in-season phase, and 12 studies were performed across both pre-season and in-season phases in combination. The duration of exposure to training sessions ranged from 3 to 45 weeks across included studies. Regarding training load variables, 20 studies assessed only internal training load variables, 4 studies examined only external training load variables, and 16 studies assessed internal and external load variables in combination. Studies were allocated into five separate groups pertaining to the type of performance outcome measured, including endurance (33 studies), strength (3 studies), speed (11 studies), power (17 studies), and body composition (2 studies).

**Table 4:** Methodological approaches of included studies

Study	Period	Weeks	Training load measures	Outcomes
Gorostiaga et al. [54] ^1^	Pre-season, first half of season	45	Total strength training time (min), endurance and ball exercise training time at low intensity (min), endurance and ball exercise training time at high intensity (min)	Standing throwing velocity (m.s^− 1^), power at 125% of body mass half-squat (W), velocity at blood lactate concentration of 3 mmol.L^− 1^ (m.s^− 1^)
Stagno et al. [55]	Pre-season, first half of season	8	Mean weekly TRIMP modified (AU), mean weekly time spent in high-intensity activity (min)^2^	VO_2max_ (ml.kg^− 1^.min^− 1^), V_OBLA_ (km.h^− 1^)
Granados et al. [58] ^1^	Pre-season, first half of season	45	Competition and training time (min)	Fat free mass (kg), velocity at 30% of maximal repetition bench press (m.s^− 1^)
Castagna et al. [79]	Pre-season	6	Time spent in high-intensity activitiy^3^ (%)	V_LT_ (km.h^− 1^), V_OBLA_ (km.h^− 1^)
Akubat et al. [93]	First half of season	6	Mean weekly sRPE (AU), bTRIMP (AU), iTRIMP (AU), Team TRIMP (AU)	V_LT_ (km.h^− 1^), V_OBLA_ (km.h^− 1^), HR_LT_ (beats.min^− 1^), HR_OBLA_ (beats.min^− 1^)
Castagna et al. [94]	Pre-season	8	Time spent in high-intensity activitiy^3^ (%)	VO_2max_ (ml.kg^− 1^.min^− 1^), Yo-Yo IRT (Level 1) distance (m), V_LT_ (km.h^− 1^), V_OBLA_ (km.h^− 1^)
Manzi et al. [74]	Pre-season	8	Mean weekly iTRIMP (AU)	VO_2max_ (ml.kg^− 1^.min^− 1^), V_OBLA_ (km.h^− 1^), VO_2VT_ (km.h^− 1^), Yo-Yo IRT (Level 1) distance (m)
Arcos et al. [95]	First half of season	9	Total added time training and matches (min), sum sRPEmus (AU)	CMJ height (cm), 15-m sprint time (s)
Arcos et al. [92]	Pre-season, first half of season	9	sRPEresp (AU), sRPEmusc (AU), sumRPEresp (AU), sumRPEmusc (AU), training volume (min)	CMJ height (cm), CMJ arm swing height (cm), CMJ dominant leg height (cm), CMJ non-dominant leg height (cm), 5-m sprint time (s), 15-m sprint time (s), velocity at blood lactate concentration of 3 mmol.L^− 1^ (km.h^− 1^), blood lactate at 12 km.h^− 1^ (mmol.L^− 1^), blood lactate at 13 km.h^− 1^ (mmol.L^− 1^)
Gil-Rey et al. [60]	First half of season	9	Total accumulated sRPEresp (AU), total accumulated sRPEmus (AU), training and match volume (AU), sRPEresp (AU), sRPEmusc (AU)	Time to exhaustion (min), 5-m sprint time (s), 15-m sprint time (s), CMJ height (cm), CMJ arm swing height (cm)
Nakamura et al. [59]	First half of season	9	Weekly sRPE (AU)	SJ height (cm), CMJ height (cm), JS (W), 5-m sprint velocity (m.s^− 1^), 10 m sprint velocity (m.s^− 1^), 20-m sprint velocity (m.s^− 1^)
Malone et al. [48]	First half of season	20	Weekly iTRIMP (AU)	VO_2max_ (ml.kg^− 1^.min^− 1^), Yo-Yo IRT (Level 1) distance (m), Yo-Yo IRT (Level 2) distance (m), V_LT_ (km.h^− 1^), V_OBLA_ (km.h^− 1^)
Arcos et al. [89]	Pre-season, first half of season	32	sRPEresp (AU), sRPEmusc (AU), sumRPEresp (AU), sumRPEmusc (AU), training volume (min)	CMJ height (cm), CMJ arm swing height (cm), 5-m sprint time (s), 15-m sprint time (s), running velocity with a lactate of 3 mmol.L^− 1^

VO_2max_ (maximal oxygen uptake), V_OBLA_ (velocity at a blood lactate concentration of 4 mmol.L^− 1^), V_LT_ (velocity at a blood lactate concentration of 2 mmol.L^− 1^), iTRIMP (individualized training impulse), sRPE (session rating of perceived exertion), HR_LT_ (heart rate at a blood lactate concentration of 2 mmol.L^− 1^), HR_OBLA_ (heart rate at blood lactate concentration of 4 mmol.L^− 1^), Yo-Yo IRT (Yo-Yo Intermittent Recovery Test), VO_2VT_ (ventilatory threshold), sRPEmus (session rating of perceived exertion local-muscular), CMJ (countermovement jump), sRPEresp (session rating of perceived exertion respiratory), sumRPEmusc (sum of all muscular perceived efforts), sumRPEresp (sum of all respiratory perceived efforts), SJ (squat jump), JS (jump squat), sum RPE (sum of all rating of perceived efforts), Edwards’_s_ (Edward’s training impulse according to heart rate reserve), V_IFT_ (final velocity during intermittent fitness testing), sRPE_RT_ (session rating of perceived exertion resistance training), sRPE_COND_ (session rating of perceived exertion conditioning), sRPE_SK_ (session rating of perceived exertion skills), COD (change-of-direction), Mognoni’s_LA_ (Mognoni’s continuous test blood lactate concentration), HIT_LA_ (high-intensity intermittent running test blood lactate concentration), eTRIMP (Edward’s training impulse), 17HSD (high-speed distance covered >17 km.h^− 1^), VHSD (very high-speed distance covered >21 km.h^− 1^), MAS (maximal aerobic speed), MSS (maximal sprint speed), luTRIMP (Lucia’s training impulse), bTRIMP (Bannister’s training impulse), iHSD (individualized high-speed distance), 15HSD (high-speed distance >15 km.h^− 1^), 18HSD (high-speed distance >18 km.h^− 1^), V_VO2max_ (velocity at VO_2max_), PT (peak torque), V_IFT_ (final velocity during the 30 − 15 Intermittent Fitness Test), HIR (high-intensity running distance covered >14.4 km.h^− 1^), VHIR (very high-intensity running distance covered >19.8 km.h^− 1^), RSSA (repeated-sprint shuttle ability), ASR (anaerobic sprint reserve), gTRIMP (Stagno individualized training impulse), RSA (repeated-sprint ability test), HSRD (high-speed running distance), SD (sprint distance), VHSRD (very high-speed running distance), V_Vavemal_ (final velocity reached in the Vavemal test), RM (maximal repetition). ^1^The correlations were performed for specific periods of training; ^2^High-intensity activity corresponds to zones 4 (86–92%) and 5 (93–100%) of maximal heart rate. The training type of zones 4 and 5 represent OBLA (blood lactate concentration at 4 mmol.L^− 1^) and maximal training, respectively; ^3^Heart rates corresponding to blood lactate concentration >4 mmol.L^− 1^; ^4^ HR was categorized as low intensity (≤ HR at blood lactate concentration of 2 mmol·L^− 1^), medium intensity (between HR corresponding to blood lactate concentration of 2 and 4 mmol·L^− 1^), and high intensity (≥ HR at blood lactate concentration of 4 mmol·L^− 1^); ^5^High intensity HR zone corresponds to 90–100% of maximal heart rate; 14-19HSRD (high-speed running distance 14–19 km.h^− 1^), 20VHSRD (very high-intensity running distance covered >20.0 km.h^− 1^); HR_MEAN_ (mean heart rate), 13LIRD (low-intensity running distance < 13 km.h^− 1^), 13-19HIRD (high-intensity running distance 13–19 km.h^− 1^), 19VHIRD (very high-intensity running distance >19 km.h^− 1^); ^#^In the study Quan et al. [106] zone 2 was defined between 7.0–14.99 km.h^− 1^

### Results of Individual Studies and meta-analysis

#### Endurance

Supplementary material 2 presents the correlations between training loads and changes in endurance performance across individual studies. Within the endurance performance domain, studies were further categorized into two groups, including: (i) indicators associated with aerobic endurance (VO_2max_, Yo-Yo Intermittent Recovery Test performance, time to exhaustion, final velocity during intermittent fitness testing (V_IFT_), maximal aerobic speed, shuttle-run test distance, and final velocity reached in last stage of the Vavemal test (V_Vavemal_)); and (ii) indicators associated with blood lactate concentration (velocity at blood lactate concentration of 3 mmol.L^− 1^, speed at 2, 3, and 4 mmol.L^− 1^, HR at 2 and 4 mmol.L^− 1^, blood lactate concentration at 12 and 13 km.h^− 1^, Mognoni’s continuous test blood lactate concentration, and high-intensity intermittent running test blood lactate concentration).

**Fig. 3:**
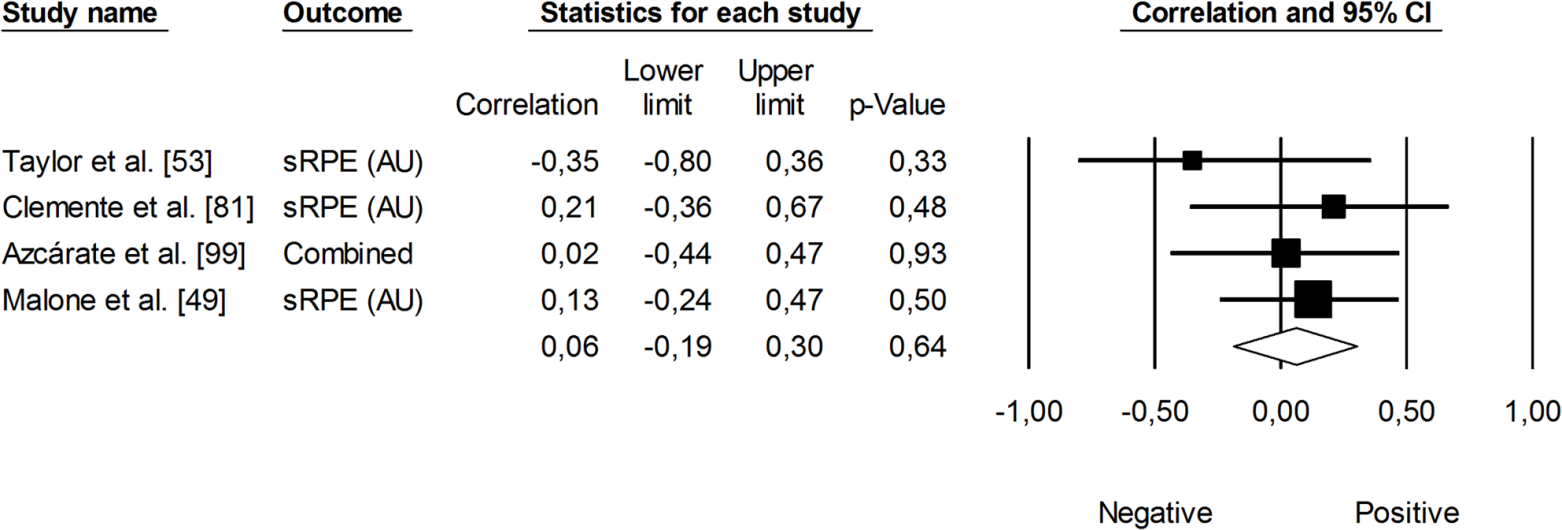
Meta-analysis between session rating of perceived exertion and changes in VO_2max_. sRPE (session rating of perceived exertion), VO_2max_ (maximal oxygen uptake). Azcárate et al. [99] reported correlations for match sRPE local-muscular and training sRPE respiratory

**Fig. 4:**
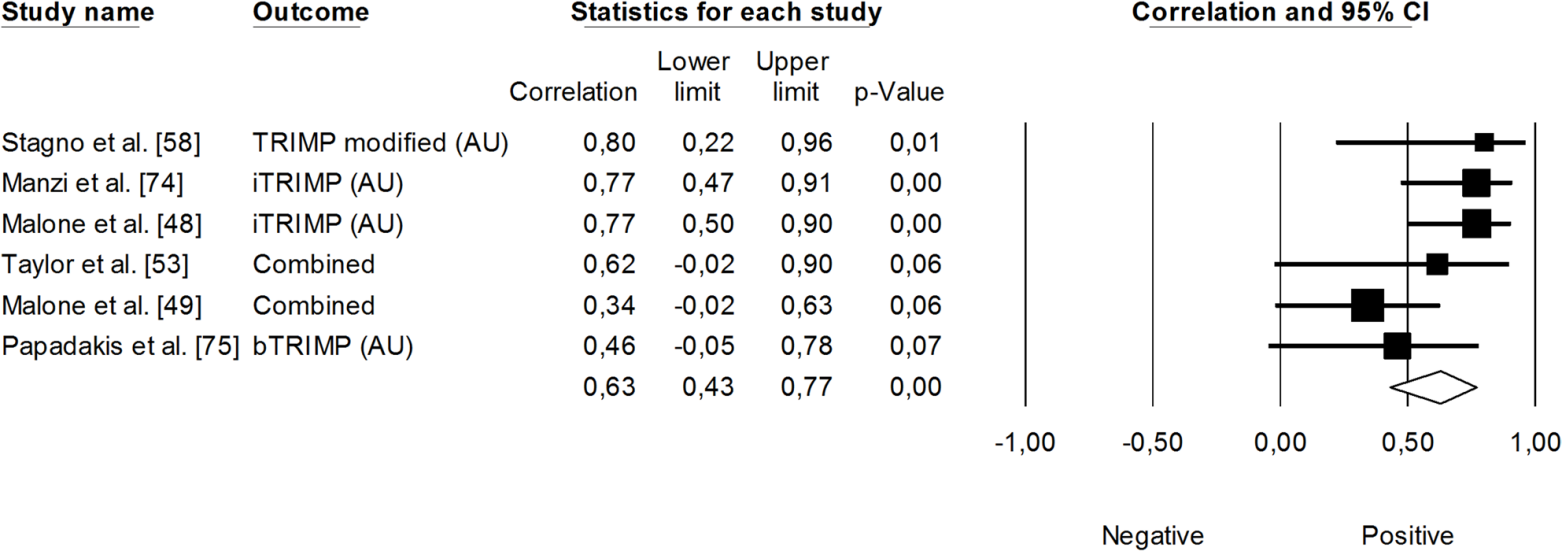
Meta-analysis between training impulse and changes in VO_2max_. TRIMP (training impulse), iTRIMP (individualized training impulse), bTRIMP (Bannister’s training impulse). Taylor et al. [53] reported correlations for four TRIMPs (individualized, Lucia’s, Edwards’, and Bannister’s methods). Malone et al. [49] reported correlations for five TRIMPs (individualized, Lucia’s, Edwards’, Bannister’s, and Stagno’s methods). The separate correlation coefficients reported in these studies were combined (i.e., averaged) within each of them

**Fig. 5:**
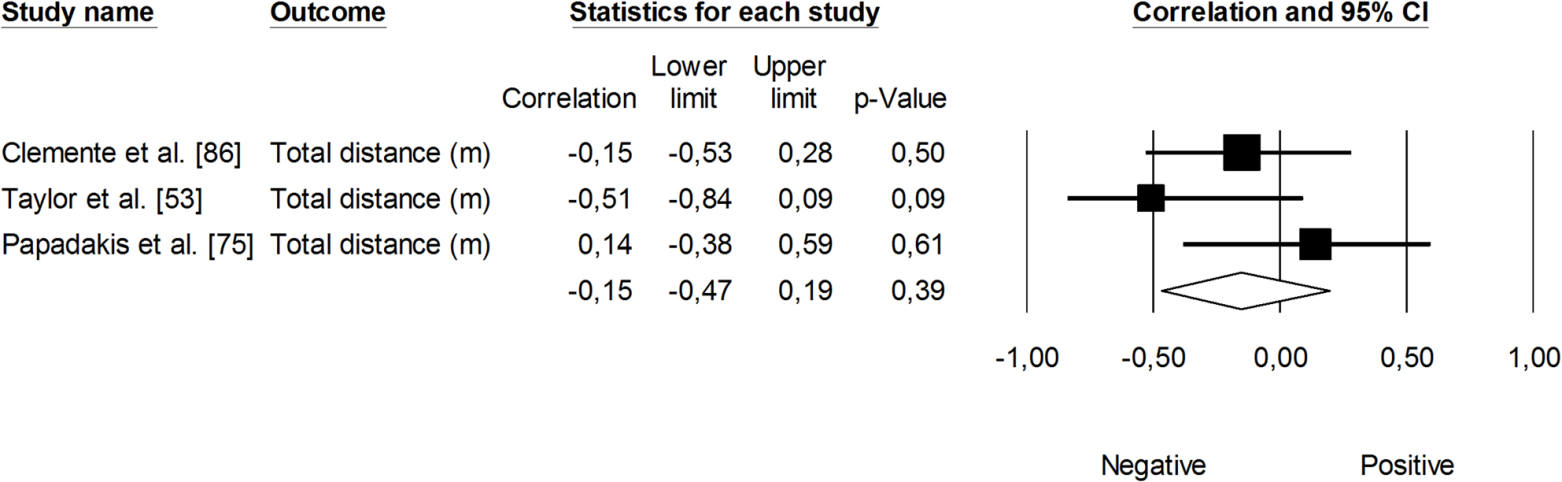
Meta-analysis between total distance covered and changes VO_2max_

The correlation between session rating of perceived exertion (RPE × session duration; sRPE) and changes in VO_2max_ was trivial (*r* = 0.06, 95% CI: -0.19 to 0.30, *p* = 0.64). The impact of heterogeneity among the correlation coefficients was low (*I²* <5%) (Fig. 3). In contrast, the overall correlation coefficient between HR-based TRIMP variables — combining exercise volume and intensity — and changes in VO_2max_ was large (*r* = 0.63, 95% CI: 0.42 to 0.77, *p* < 0.05), with moderate heterogeneity across studies (*I²* = 36%). One trimmed study was identified; however, the adjusted correlation coefficient for random effects remained unchanged (*r* = 0.60, 95% CI: 0.42 to 0.74) (Fig. 4). Additionally, total distance covered was negatively associated with changes in VO_2max_, though this finding was not statistically significant (*r* = -0.15, 95% CI: -0.47 to 0.19, *p* = 0.39). The impact of heterogeneity was low (*I²* = 24%) (Fig. 5).

**Fig. 6:**
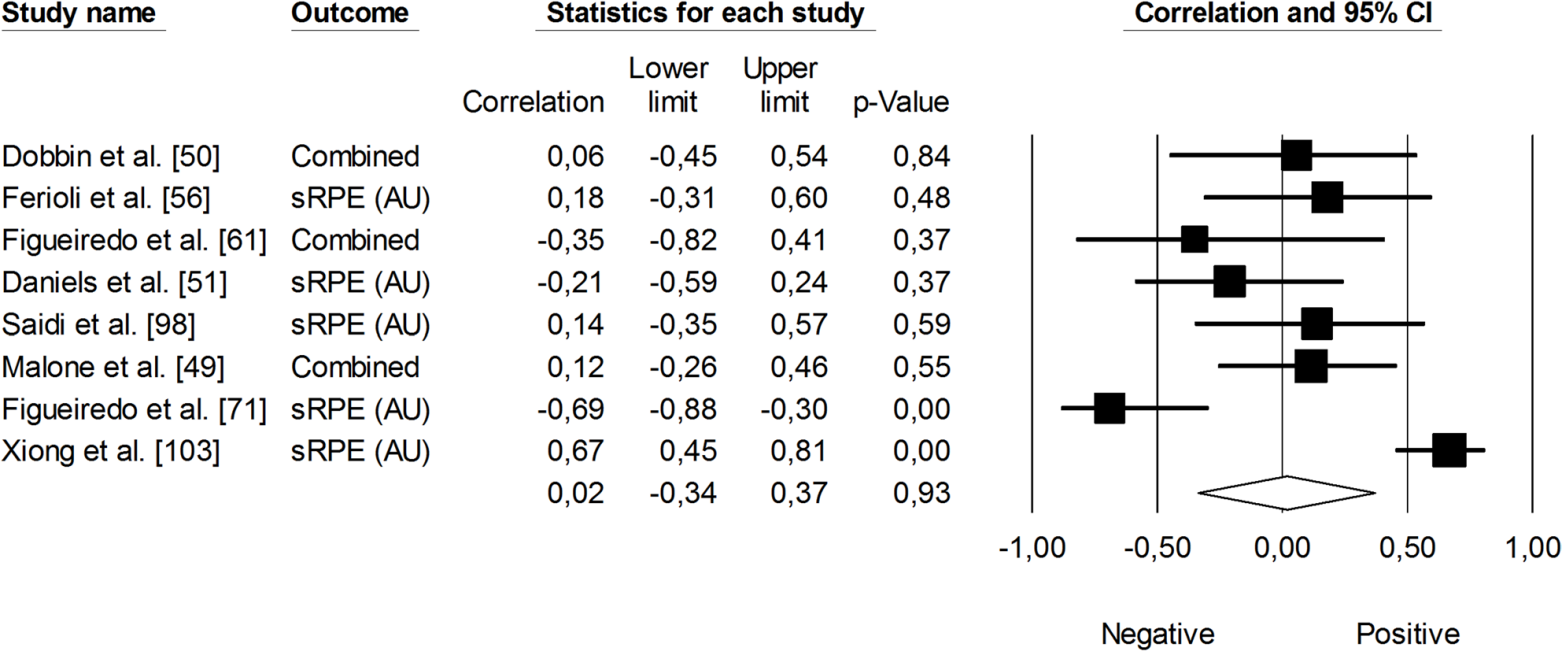
Meta-analysis between session rating of perceived exertion and changes in Yo-Yo Intermittent Recovery Test performance. Legend: sRPE (session rating of perceived exertion). Dobbin et al. [50] reported correlations for sRPE resistance training, sRPE conditioning, sRPE skills, and total sRPE. Figueiredo et al. [61] reported correlations for weekly sRPE separately in two age groups (under-15 years and under-17 years). Malone et al. [49] reported correlations for Yo-Yo Intermittent Recovery Test performance separately using Level 1 and Level 2 protocols. The separate correlation coefficients reported in these studies were combined (i.e., averaged) within each of them

**Fig. 7:**
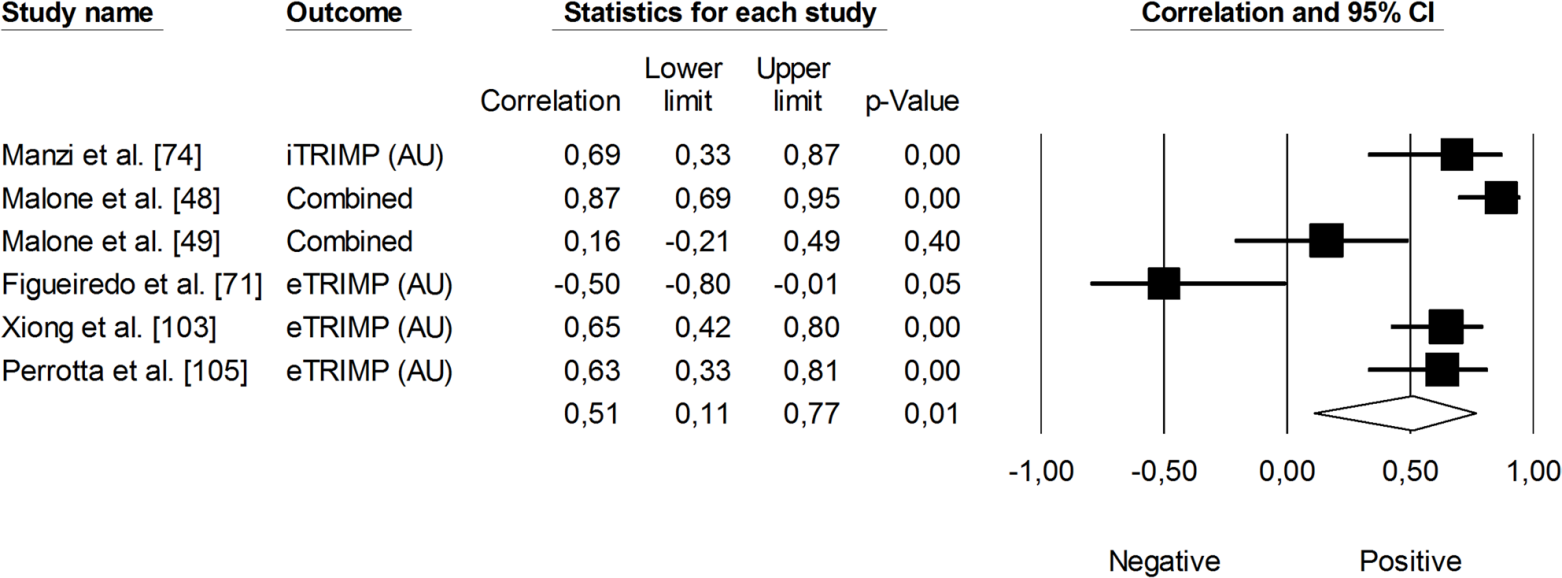
Meta-analysis between training impulse and changes in Yo-Yo Intermittent Recovery Test performance. Legend: iTRIMP (individualized training impulse). Malone et al. [48] and Malone et al. [49] reported correlations for Yo-Yo Intermittent Recovery Test performance separately using Level 1 and Level 2 protocols. The separate correlation coefficients reported in these studies were combined (i.e., averaged) within each of them

Regarding changes in Yo-Yo Intermittent Recovery Test (Yo-Yo IRT) performance, which includes both Level 1 and Level 2 protocols, the correlation with sRPE was not statistically significant (*r* = 0.02, 95% CI: -0.34 to 0.37, *p* = 0.96). The heterogeneity was high (*I²* = 79%). No trimmed studies were noted (Fig. 6). The correlation between TRIMP variables and changes in Yo-Yo IRT performance was positive and moderate in magnitude (*r* = 0.51, 95% CI: 0.11 to 0.77, *p* < 0.24). However, the impact of heterogeneity among the studies was high (*I²* = 85%) (Fig. 7).

**Fig. 8:**
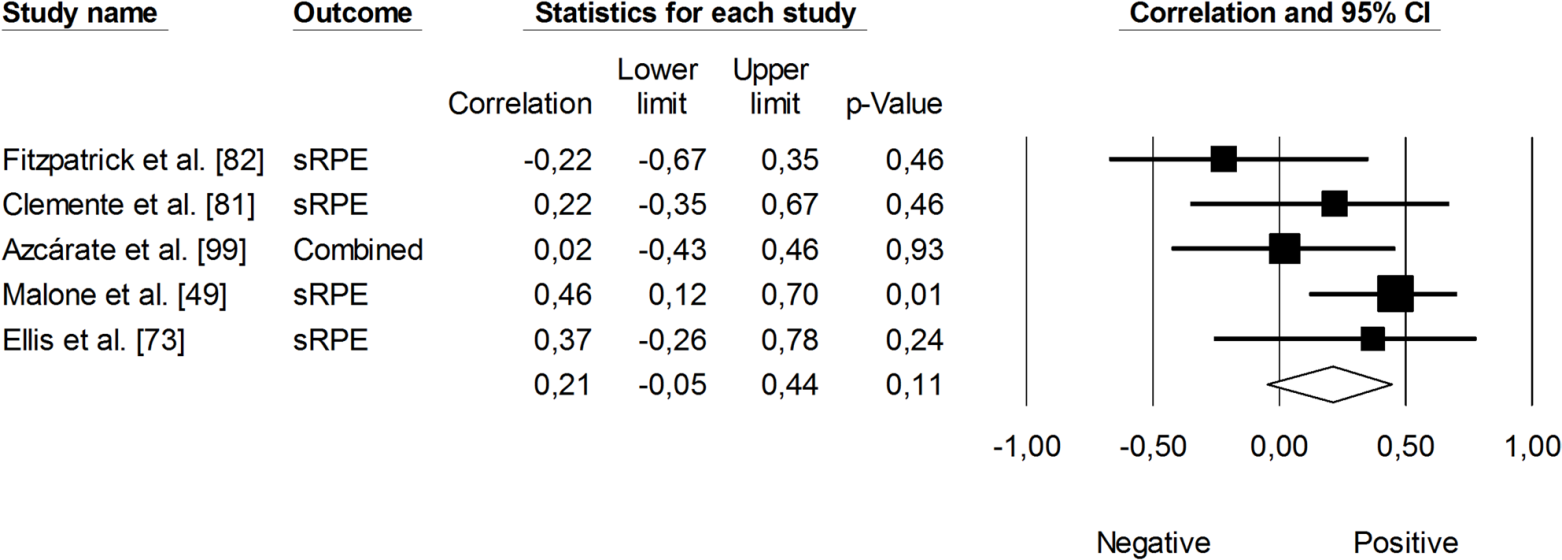
Meta-analysis between session rating of perceived exertion and changes in maximal aerobic speed. sRPE (session rating of perceived exertion). Azcárate et al. [99] reported correlations for match sRPE local-muscular and training sRPE respiratory. The separate correlation coefficients reported in this study were combined (i.e., averaged)

Figure 8 illustrates the meta-analysis examining the relationship between sRPE and changes maximal aerobic speed. The overall correlation coefficient was small and not statistically significant (*r* = 0.21, 95% CI: -0.05 to 0.44, *p* = 0.48). The impact of heterogeneity among the studies was low (*I²* = 22%).

**Fig. 9:**
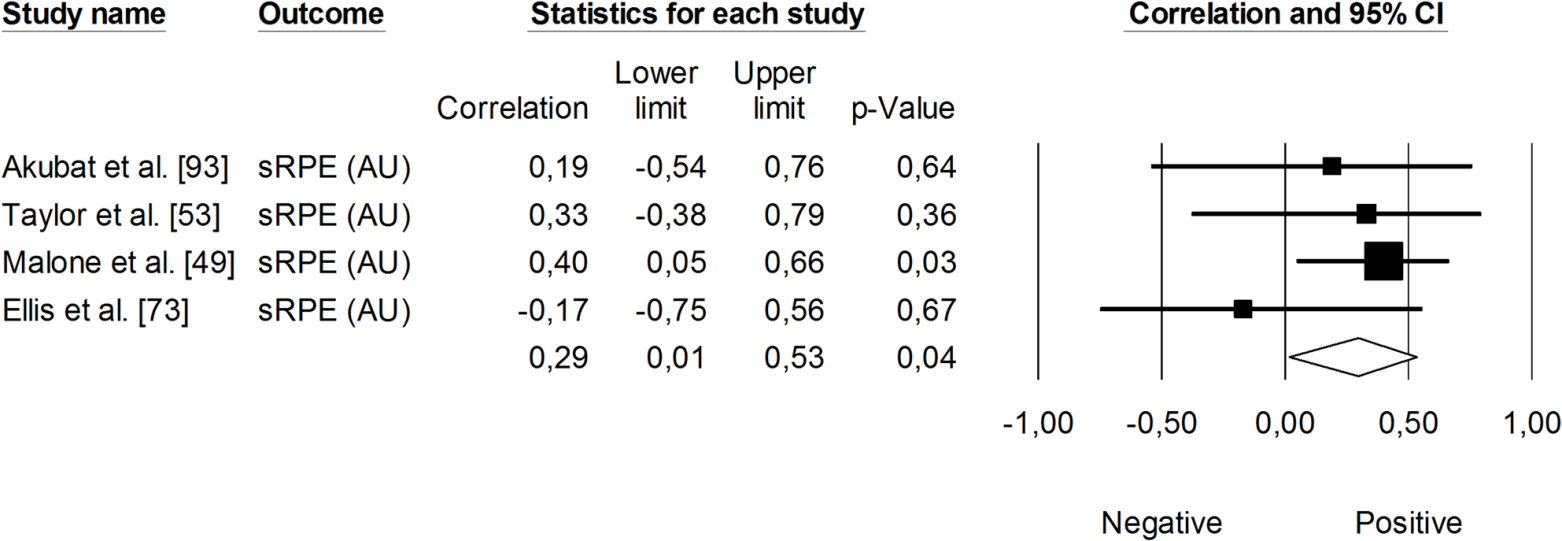
Meta-analysis between session rating of perceived exertion and changes in velocity at a blood lactate concentration of 2 mmol·L^− 1^). sRPE (session rating of perceived exertion)

**Fig. 10:**
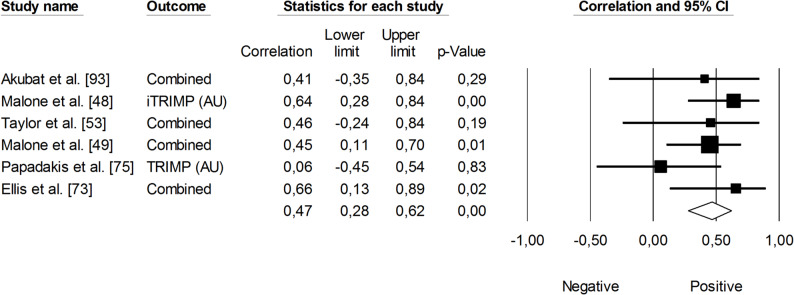
Meta-analysis between heart rate-based training impulse (TRIMP) and changes in velocity at a blood lactate concentration of 2 mmol·L^− 1^ (V_LT_). Legend: iTRIMP (individualized training impulse). Akubat et al. [93] reported correlations for four TRIMPs (Bannister’s, Edward’s, team, and individualized methods). Taylor et al. [53] and Ellis et al. [73] each reported correlations for four TRIMPS (individualized, Lucia’s, Edwards’, and Bannister’s). The separate correlation coefficients reported in these studies were combined (i.e., averaged) within each of them

**Fig. 11:**
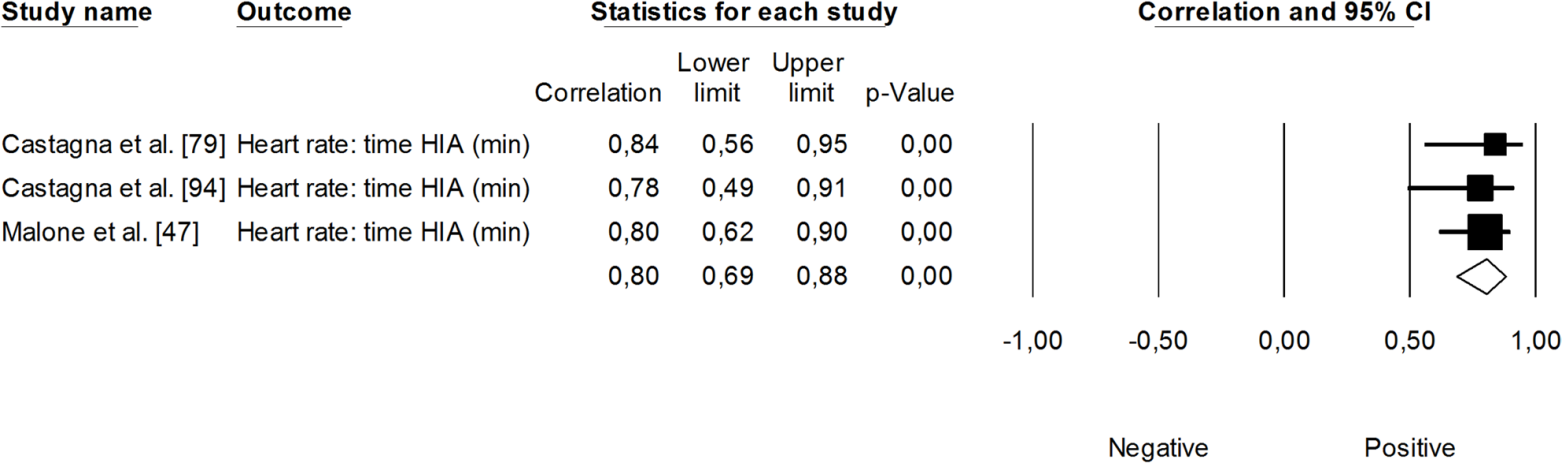
Meta-analysis between time spent at high heart rate intensities and changes in velocity at a blood lactate concentration of 2 mmol·L^− 1^. HIA (high-intensity activity)

**Fig. 12:**
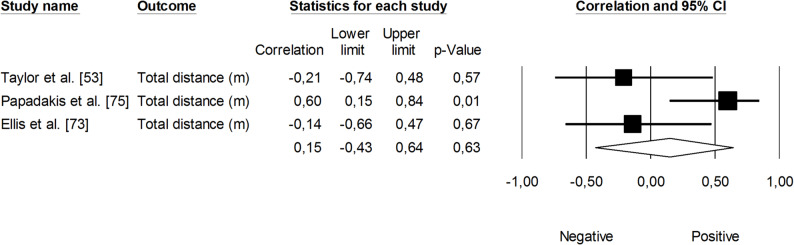
Meta-analysis between total distance covered and changes in velocity at a blood lactate concentration of 2 mmol.L^− 1^

The correlation between sRPE and changes in velocity at a blood lactate concentration of 2 mmol·L⁻¹ (V_LT_) was significant and small (*r* = 0.29, 95% CI: 0.01 to 0.53, *p* = 0.01). The impact of heterogeneity across studies was low (*I²* <5%). Two trimmed studies were identified, leading to an adjusted effect size that increased from small to moderate (*r* = 0.39, 95% CI: 0.14 to 0.58) (Fig. 9). The overall correlation between TRIMP variables and changes in V_LT_ was significant and moderate (*r* = 0.47, 95% CI: 0.28 to 0.52, *p* < 0.05). One trimmed study was identified for random effects, resulting in a correlation of *r* = 0.53 (95% CI: 0.33 to 0.69). The impact of heterogeneity remained low (*I²* <5%) (Fig. 10). Additionally, time spent in high-intensity activities (i.e., >4 mmol.L^− 1^) had a significant, very large, positive correlation with changes in V_LT_ (*r* = 0.80, 95% CI: 0.69 to 0.88, *p* < 0.05), as shown in Fig. 11. The heterogeneity across studies was low (*I²* <5%). Finally, Fig. 12 illustrates the correlations between total distance covered and V_LT_, with a trivial overall correlation found (*r* = 0.15, 95% CI: -0.43 to 0.64, *p* = 0.63). A moderate impact of heterogeneity was apparent across studies (*I²* = 63%).

**Fig. 13:**
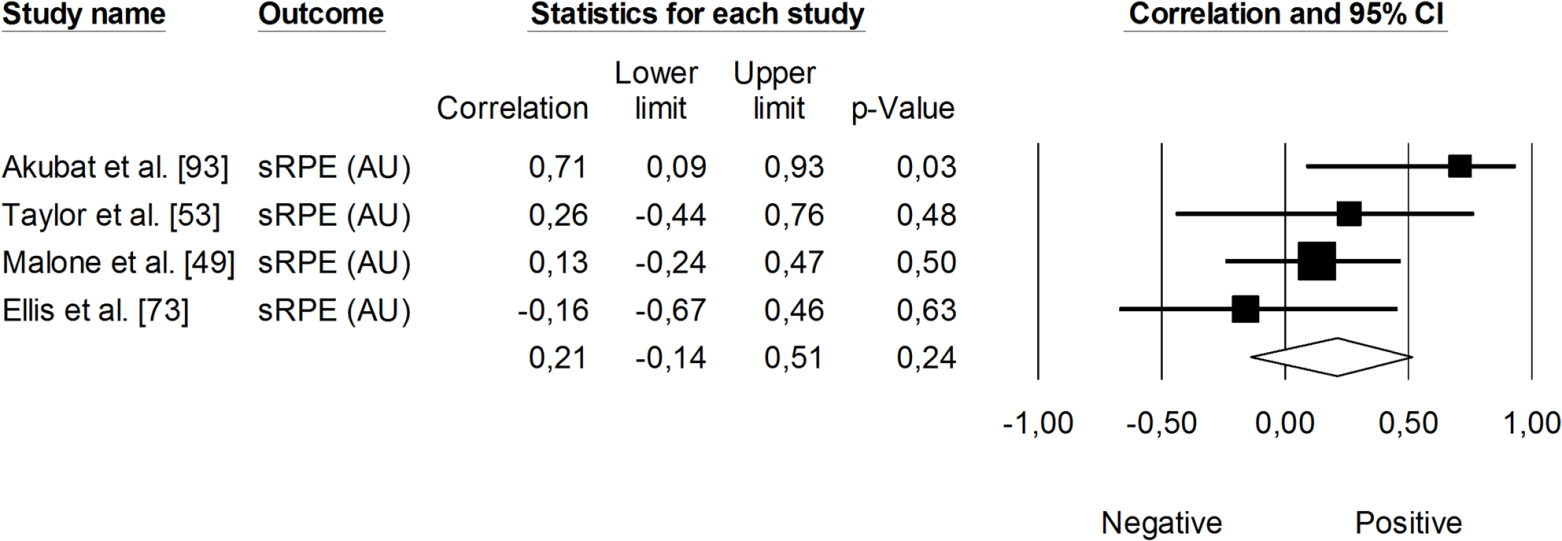
Meta-analysis between session rating of perceived exertion and changes in velocity at a blood lactate concentration of 4 mmol·L^− 1^. sRPE (session rating of perceived exertion)

**Fig. 14:**
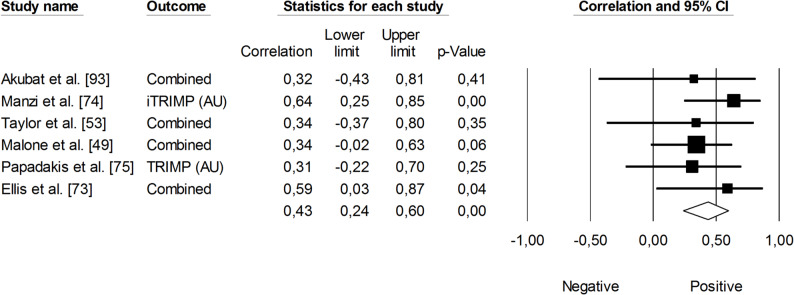
Meta-analysis between heart rate-based training impulse (TRIMP) and changes in velocity at a blood lactate concentration of 4 mmol.L^− 1^. iTRIMP (individualized training impulse. Akubat et al. [93] reported correlations for four TRIMPs (Bannister’s, Edward’s, team, and individualized methods). Taylor et al. [53], and Ellis et al. [73] reported correlations for four TRIMPS (individualized, Lucia’s, Edwards’, and Bannister’s methods). Malone et al. [49] reported correlations for five TRIMPs (individualized, Lucia’s, Edwards’, Bannister’s, and Stagno’s methods). The separate correlation coefficients reported in these studies were combined (i.e., averaged) within each of them

**Fig. 15:**
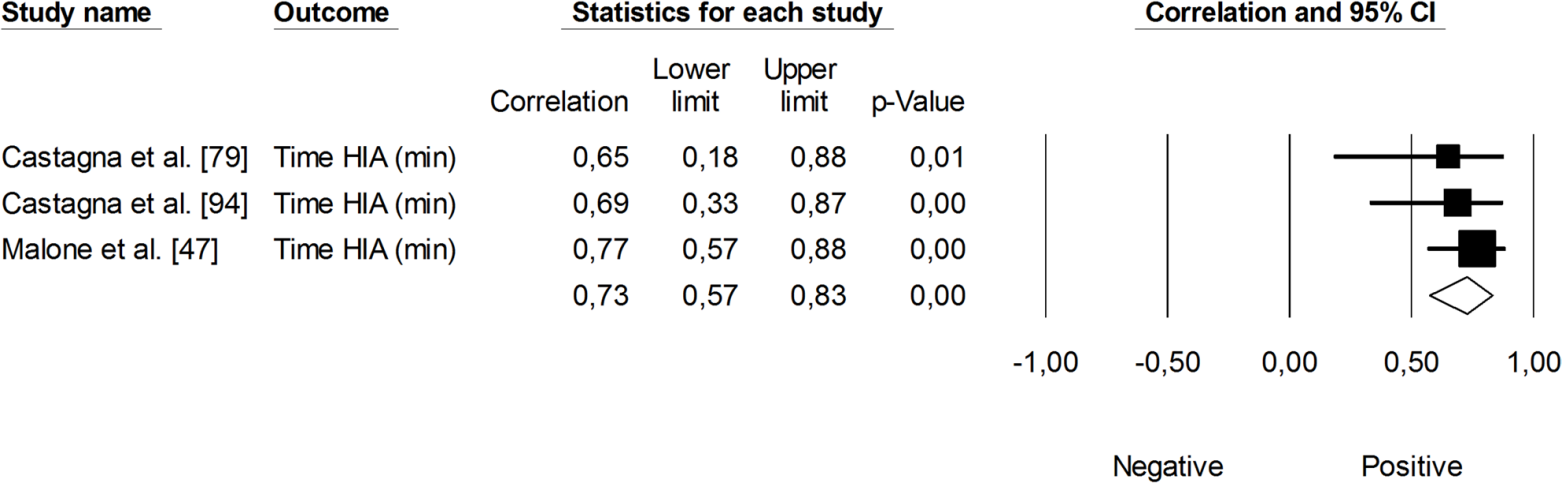
Meta-analysis between time spent at high heart rate intensities and changes in velocity at a blood lactate concentration of 4 mmol.L^− 1^. HIA (high-intensity activity)

**Fig. 16:**
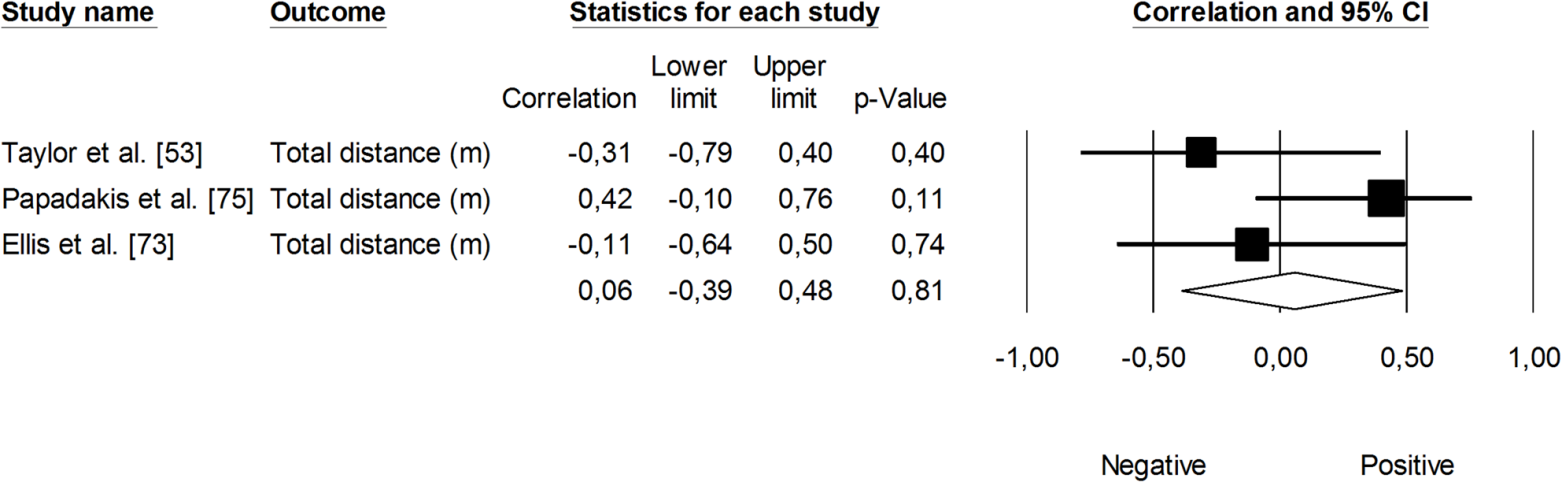
Meta-analysis between total distance covered and changes in velocity at a blood lactate concentration of 4 mmol.L^− 1^

Changes in velocity at a blood lactate concentration of 4 mmol·L⁻¹ (V_OBLA_) were analyzed for their correlations with sRPE (Fig. 13), TRIMP variables (Fig. 14), HR (Fig. 15), and total distance covered (Fig. 16). A small and non-significant correlation was found between sRPE and changes in V_OBLA_ (*r* = 0.21, 95% CI: -0.14 to 0.51, *p* = 0.24), with moderate impact of heterogeneity across studies (*I²* = 28%). The overall correlation between TRIMP variables and changes in V_OBLA_ was moderate (*r* = 0.43, 95% CI: 0.24 to 0.60, *p* < 0.01). No trimmed studies were noted, and the impact of heterogeneity was low (*I²* <5%). A very large correlation was observed between time spent in high-intensity activities indicated via HR assessment and changes in V_OBLA_ (*r* = 0.73, 95% CI: 0.57 to 0.83, *p* < 0.05). The impact of heterogeneity among the correlation coefficients was low (*I²* <5%). The adjusted overall correlation remained unchanged in magnitude (*r* = 0.77, 95% CI: 0.66 to 0.85). In contrast, total distance covered was not significantly associated with changes in V_OBLA_ (*r* = 0.06, 95% CI: -0.39 to 0.48, *p* = 0.81). The impact of heterogeneity for this analysis was moderate (*I²* = 38%).

#### Power Output

**Fig. 17:**
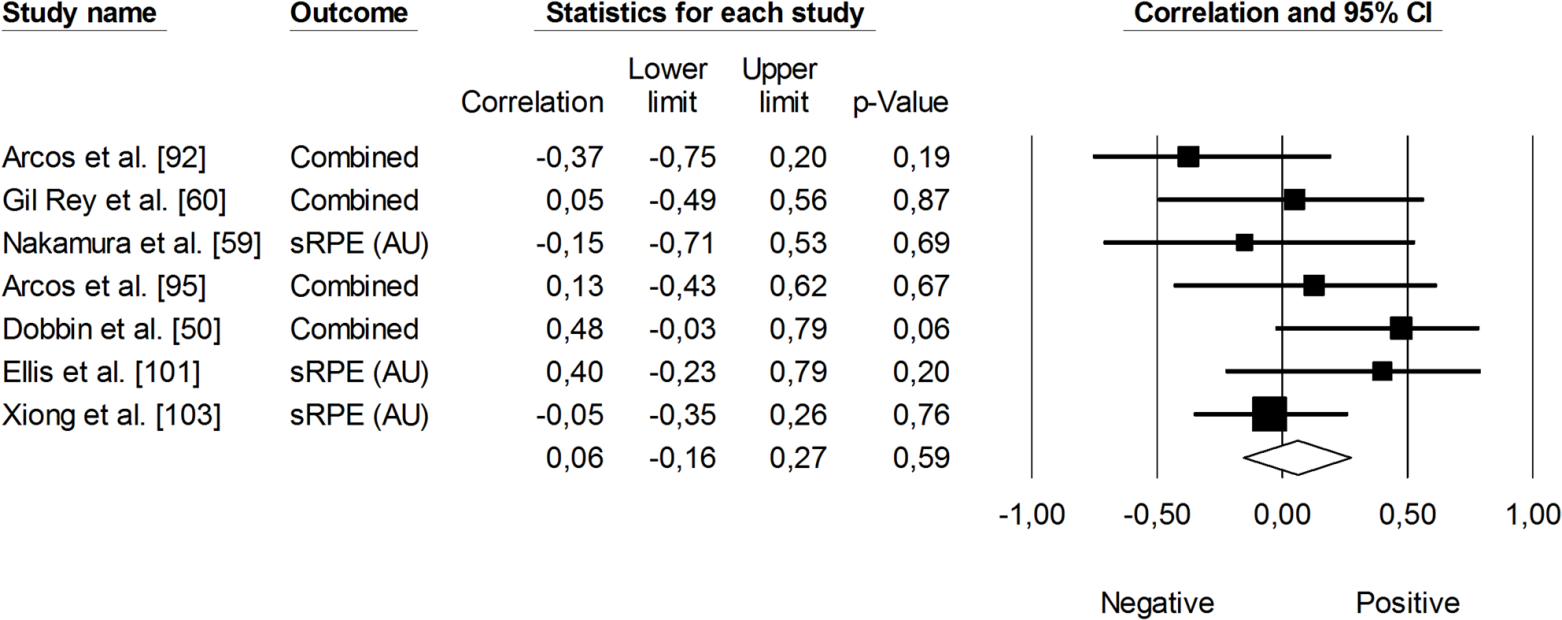
Meta-analysis between session rating of perceived exertion and jump performance. sRPE (session rating of perceived exertion). Arcos et al. [92] and Arcos et al. [95] reported correlations for sRPE respiratory and sRPE conditioning. Gil Rey et al. [60] reported correlations for sRPE respiratory and sRPE conditioning using countermovement jump and countermovement jump with arm swing protocols. Dobbin et al. [50] reported correlations for sRPE resistance training, sRPE conditioning, sRPE skills, and total sRPE. The separate correlation coefficients reported in these studies were combined (i.e., averaged) within each of them

Supplementary material 3 summarizes the correlations between training load and changes in power output across individual studies. The correlation between sRPE and changes in countermovement jump (CMJ) power output was small and non-significant (*r* = 0.06, 95% CI: -0.16 to 0.27, *p* = 0.59). The impact of heterogeneity across studies was low (*I²* = 14%) (Fig. 17).

#### Strength

The relationships between training load and changes in strength output reported in individual studies are summarized in supplementary material 4. Due to the limited data representing correlations between training load and this performance measure (three studies) using different methodological protocols and outcome measures, a meta-analysis was not able to be conducted.

#### Speed

**Fig. 18:**
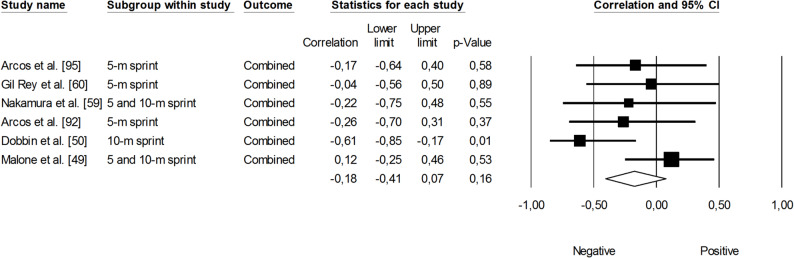
Meta-analysis between session rating of perceived exertion and linear sprint performance *≤* 10 m. sRPE (session rating of perceived exertion). Arcos et al. [93] and Arcos et al. [96] reported correlations for sRPE respiratory and sRPE conditioning. Gil Rey et al. [60] reported correlations between sRPE respiratory and sRPE conditioning. Nakamura et al. [59] and Malone et al. [49] reported correlations using 5- and 10-m sprint protocols. Dobbin et al. [50] reported correlations for sRPE resistance training, sRPE conditioning, sRPE skills, and total sRPE. The separate correlation coefficients reported in these studies were combined (i.e., averaged) within each of them. A negative correlation indicates an increased training load was associated with better sprint performance

**Fig. 19:**
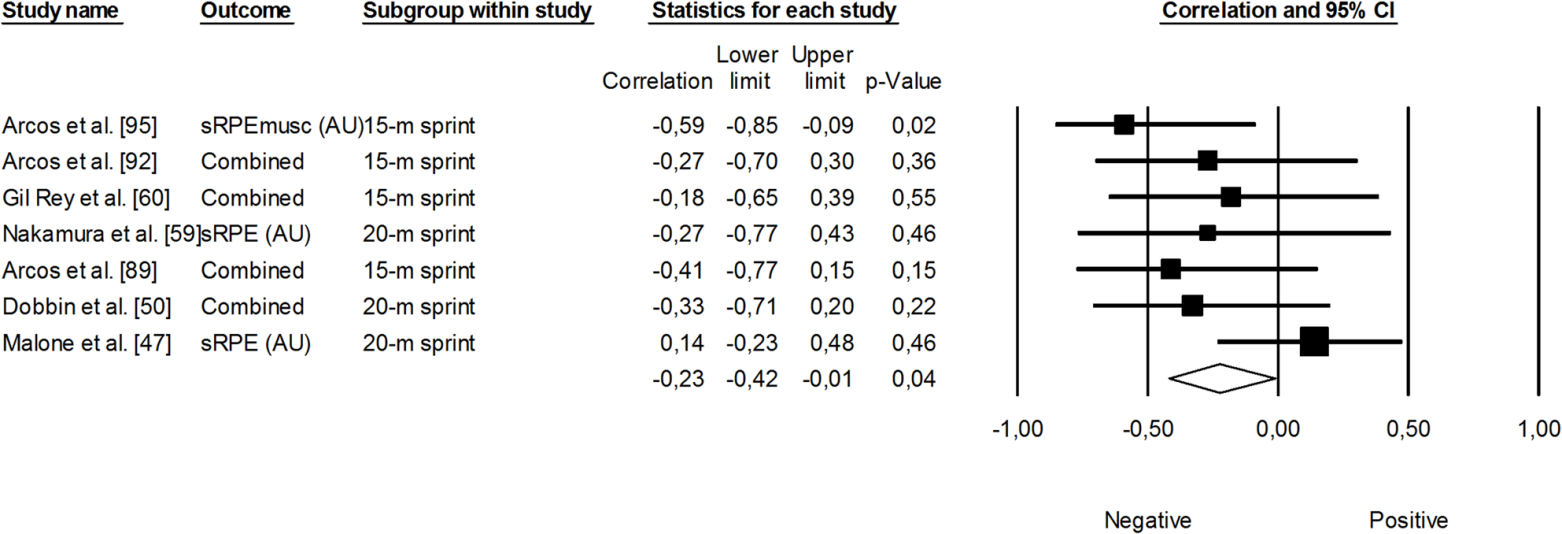
Meta-analysis between session rating of perceived exertion and linear sprint performance >10 m. sRPEmusc (session rating perceived exertion local-muscular), sRPE (session rating perceived exertion). Arcos et al. [92] and Arcos et al. [95] reported correlations for sRPE respiratory and sRPE conditioning. Gil Rey et al. [60] reported correlations between sRPE respiratory and sRPE conditioning. Dobbin et al. [50] reported correlations for sRPE training, sRPE conditioning, sRPE skills and total sRPE. The separate correlation coefficients reported in these studies were combined (i.e., averaged) within each of them. A negative correlation indicates an increased training load was associated with better sprint performance

Supplementary material 5 presents the relationships reported between training load and changes in speed across individual studies. Within the speed performance domain, studies were further categorized into two groups, including: (i) linear speed; and (ii) change-of-direction speed. Since only one study examined changes in change-of-direction speed, meta-analyses were only conducted across studies assessing linear speed. Linear speed test protocols were categorized as those ≤ 10 m and those >10 m for meta-analyses [62]. The correlation between sRPE and changes in linear sprint performance across *≤* 10 m was not significant, small, and negative (*r* = -0.18, 95% CI: -0.41 to 0.07, *p* = 0.16). The impact of heterogeneity was small across studies (*I*^*2*^ = 22%) (Fig. 18). The correlation between sRPE and changes in linear sprint performance across >10 m was significant, small, and negative (*r* = -0.23, 95% CI: -0.42 to -0.01, *p* = 0.04). The impact of heterogeneity across studies was small (*I*^*2*^ = 9%). The removal of four trimmed studies adjusted the overall correlation coefficient to a trivial magnitude (*r* = -0.03, 95% CI: -0.26 to 0.19) (Fig. 19).

#### Body Composition

The relationships between training load and changes in body composition reported in individual studies are summarized in Supplementary material 6. Due to the limited data representing correlations between training load and changes in body composition (two studies), a meta-analysis was not able to be conducted.

### Certainty of Evidence

As per GRADE guidelines, non-randomized studies start at low certainty of evidence and can be further downgraded according to five dimensions, while upgrades can only be performed in the absence of relevant downgrades. Regarding the five dimensions for downgrading, indirectness was considered low, while risk of publication bias was not assessed due to all comparisons having less than 10 studies available [38, 39]. All comparisons included in this review were downgraded by 1 level due to concerns regarding risk of bias in studies, which automatically placed all comparisons at very low certainty of evidence (i.e., the lowest possible level). However, there were additional concerns that would lead to further downgrading, were that possible. Five of 16 analyses would require downgrading by one level due to inconsistency (i.e., moderate impact of heterogeneity), while one analysis would require downgrading by two levels (high impact of heterogeneity).

Analyses would further require downgrading due to imprecision: (i) all analyses were downgraded by one level due falling well below the optimal sample size of 800 participants [46]; and (ii) nine analyses (~ 56%) were downgraded an extra level due to unclear direction of effects. Ultimately, all analyses were judged to be of very low level of certainty. Even if we treated the studies as being randomized, and therefore starting at a high level of certainty (which would be inappropriate), only six analyses (37.5%) would be judged to be of low level of certainty, with all others being rated as being of very low certainty of evidence (Table 5).

**Table 5:** GRADE assessments for the included studies in this review and meta-analysis

Correlation variables	k (*n*)	Risk of bias	Indirectness	Risk of publication bias	Inconsistency	Imprecision	Certainty of evidence
**Endurance**							
sRPE x maximal aerobic speed	5 (96)	Downgrade by 1 level (PEDro < 85%).	No downgrading	N/A	No downgrading (*I*^2^ = 22%).	Downgrade by 2 levels (< 800 participants and no clear direction of effects	⊕
sRPE x V_LT_	4 (58)	Downgrade by 1 level (PEDro < 85%).	No downgrading	N/A	No downgrading (*I*^2^ = 5%).	Downgrade by 1 level (< 800 participants)	⊕
sRPE x V_OBLA_	4 (58)	Downgrade by 1 level (PEDro < 85%).	No downgrading	N/A	Downgrade by 1 level (*I*^2^ = 28%).	Downgrade by 2 levels (< 800 participants and no clear direction of effects	⊕
sRPE x Yo-Yo (levels 1 and 2)	8 (169)	Downgrade by 1 level (PEDro < 85%).	No downgrading	N/A	Downgrade by 1 level (*I*^2^ = 79%).	Downgrade by 2 levels (< 800 participants and no clear direction of effects	⊕
Total distance x V_LT_	3 (35)	Downgrade by 1 level (PEDro < 85%).	No downgrading	N/A	Downgrade by 1 level (*I*^2^ = 63%).	Downgrade by 2 levels (< 800 participants and no clear direction of effects	⊕
Total distance x V_OBLA_	3 (35)	Downgrade by 1 level (PEDro < 85%).	No downgrading	N/A	Downgrade by 1 level (*I*^2^ = 38%).	Downgrade by 2 levels (< 800 participants and no clear direction of effects	⊕
Total distance x VO_2_max	3 (40)	Downgrade by 1 level (PEDro < 85%).	No downgrading	N/A	No downgrading (*I*^2^ = 24%).	Downgrade by 2 levels (< 800 participants and no clear direction of effects	⊕
TRIMP x V_LT_	6 (94)	Downgrade by 1 level (PEDro < 85%).	No downgrading	N/A	No downgrading (*I*^2^ = 5%).	Downgrade by 1 level (< 800 participants)	⊕
TRIMP x V_OBLA_	6 (92)	Downgrade by 1 level (PEDro < 85%).	No downgrading	N/A	No downgrading (*I*^2^ = 5%).	Downgrade by 1 level (< 800 participants)	⊕
TRIMP x VO_2_max	6 (102)	Downgrade by 1 level (PEDro < 85%).	No downgrading	N/A	Downgrade by 1 level (*I*^2^ = 36%).	Downgrade by 1 level (< 800 participants)	⊕
TRIMP x Yo-Yo (levels 1 and 2)	6 (152)	Downgrade by 1 level (PEDro < 85%).	No downgrading	N/A	Downgrade by 2 levels (*I*^2^ = 85%).	Downgrade by 2 levels (< 800 participants and no clear direction of effects	⊕
TSHIA (HR) x V_LT_	3 (62)	Downgrade by 1 level (PEDro < 85%).	No downgrading	N/A	No downgrading (*I*^2^ = 5%).	Downgrade by 1 level (< 800 participants)	⊕
TSHIA (HR) x V_OBLA_	3 (62)	Downgrade by 1 level (PEDro < 85%).	No downgrading	N/A	No downgrading (*I*^2^ = 5%).	Downgrade by 1 level (< 800 participants)	⊕
**Power**							
sRPE x CMJ power output	7 (121)	Downgrade by 1 level (PEDro < 85%).	No downgrading	N/A	No downgrading (*I*^2^ = 14%).	Downgrade by 2 levels (< 800 participants and no clear direction of effects	⊕
**Speed**							
RPE x 5- and 10-m sprint	6 (112)	Downgrade by 1 level (PEDro < 85%).	No downgrading	N/A	No downgrading (*I*^2^ = 22%).	Downgrade by 2 levels (< 800 participants and no clear direction of effects	⊕
RPE x > 10-m sprint	7 (133)	Downgrade by 1 level (PEDro < 85%).	No downgrading	N/A	No downgrading (*I*^2^ = 9%).	Downgrade by 1 level (< 800 participants)	⊕

Rules for assessments: see 'Certainty Assessment' section

CMJ: countermovement jump. *k*: number of trials. *n*: number of participants. N/A: not applicable (less than 10 studies). RPE: rating of perceived exertion. sRPE: session ratting of perceived exertion. TRIMP: training impulse. TSHIA: time spent in high-intensity activities indicated via heart rate measurement. V_LT_: velocity at a blood lactate concentration of 2 mmol.L^− 1^. ⊕: very low certainty of evidence. ⊕⊕: low certainty of evidence

## Discussion

The results of our systematic review with meta-analysis indicate that TRIMP measures are significantly and positively correlated with improvements in endurance performance (VO₂_max_, V_LT_, and V_OBLA_). Measures such as V_LT_ and V_OBLA_ appear closely linked to internal training load, showing significant positive correlations with time spent working at high-intensity HR. The sRPE also showed a small but significant correlation with V_LT_, though its association with V_OBLA_ was not significant. Moreover, sRPE did not correlate significantly with other endurance-related measures, such as VO₂max or Yo-Yo IRT performance. The meta-correlation analysis also showed that changes in power-related measures during the CMJ were not associated with training load, while linear sprint performance was significantly associated with training load using sRPE, although with small magnitude. Thus, while certain internal load measures are associated with improvements in endurance performance, changes in strength and power appear less dependent on either internal or external load measures, whereas speed showed only a small negative correlation with sRPE, suggesting limited and potentially maladaptive associations.

### Evidence Gap Map

The studies investigating the relationship between accumulated training load and physical performance adaptations in team sports highlight several important issues, particularly regarding the heterogeneity in study designs and the nature of the samples. A significant portion of these studies (approximately 65%) focused on soccer, which may limit the generalizability of the findings to other team sports, which were examined much less frequently (3–9% of studies for other sports). While internal training load can be monitored independently of the sport (e.g., through HR sensors or RPE), allowing for broader application across various team sports, external load presents challenges. The high cost of alternatives to Global Navigation Satellite Systems GNSS, such as local positioning systems, makes it more difficult to gather data from indoor team sports, which hinders the ability to generalize findings to these sports as readily as for field-based outdoor team sports like soccer. This issue is evident in the fact that studies examining indoor team sports like handball [55] and basketball [56] did not utilize external load monitoring, while studies exploring field hockey [58] and hurling [47] also opted to measure only internal training load.

The over-representation of soccer in the literature likely stems from its global popularity, widespread use of data-monitoring tools, and the prevalence of well-funded leagues that facilitate research collaborations and access to advanced equipment and expertise [63], but it also highlights a gap in the understanding of how training load affects performance in other team sports. The pooled sample of 558 players across 34 studies is relatively small given the broad scope of team sports, and the variation in sample sizes, with 68% of studies involving fewer than 20 players, raises concerns about the statistical power and the reliability of the results. Among the included studies, few employed methods to estimate the necessary a priori sample size, and there is a clear tendency to collect data based on convenience. It is important to note that convenience sampling is common in sports research, particularly in elite sports, where gaining access to teams is highly challenging. However, there is an increasing demand for more rigorous efforts to pool data from different teams to achieve greater generalizability [64]. As a result, larger sample sizes and more collaborative research efforts are now necessary to advance knowledge in this area.

Moreover, our review also verifies the underrepresentation of female players as a critical issue that warrants greater attention, particularly in light of the physiological and performance differences between sexes [55]. Biological differences such as hormonal fluctuations, muscle mass distribution, fat composition, and cardiovascular responses mean that male and female players may respond to training loads in distinct ways [65]. For instance, the menstrual cycle can influence recovery and fatigue in female players [66], factors that could alter their response to the same training loads applied to male players. This underrepresentation of females [67] means that existing findings likely do not provide insight that could guide implementation of optimal training loads for performance adaptations in females.

Another point for debate is the variability in competitive level and age among the players investigated across included studies. While most studies involved professional or elite players, nine studies investigated youth players. This discrepancy in competitive level may influence the nature of the adaptations observed, as younger players might respond differently to training loads compared to more experienced professionals due to trainability [68]. Importantly, the ability of youth athletes to accurately report perceived exertion is also constrained by cognitive development. Children and adolescents may not yet possess the formal operational cognitive skills required to consistently appraise exertion, which weakens the validity of RPE-based load monitoring in these populations [69]. Thus, both developmental stage and training history [65] complicate the interpretation of correlations, especially those involving subjective measures such as sRPE. Finally, the variation in competitive level underscores the importance of contextual factors, such as competitive demands and training environments, which may differ significantly between youth and adult players and could affect the outcomes of the training interventions.

Methodological variability in the studies also presents challenges for interpreting the results. The fact that 44% of studies were conducted during the pre-season, while only 29% were performed during the competitive season, suggests that the timing of the studies might bias the results toward pre-season adaptations. The pre-season is typically a period of intense training focused on building physical capacities, offering a greater opportunity for development following the usual rest-oriented off-season phase [70]. In contrast, in-season training primarily aims to maintain performance while effectively managing fatigue. This difference in training focus could influence the relationship between training load and changes in performance outcomes. Furthermore, the range of exposure periods, from 3 [71] to 45 [54] weeks across studies, introduces another layer of complexity, as the length of the training period could affect the magnitude of adaptations. Studies with shorter exposure periods might fail to capture the cumulative effects of training load over time, while longer studies may be better suited to assess chronic adaptations.

Finally, the diversity of performance outcomes assessed across studies somewhat restricted the specificity able to be obtained when synthesizing the findings. For instance, aerobic adaptations might occur more rapidly than improvements in speed attributes [72]. Additionally, the fact that 18 studies assessed internal training load, 12 combined internal and external loads, and only four focused exclusively on external load highlights a lack of consistency in the operationalization of training load across studies. This variation could affect the ability to compare findings across studies and raises questions about which training load measures are most correlated to specific performance adaptations. External load reflects the physical outputs during activities prescribed to the athlete, which is straightforward to measure and apply in practice [4]. However, internal load captures the athlete’s psychophysiological response to these activities and ultimately dictates the adaptations that occur [3].

### Adaptations in Physiological and Performance-related Endurance Outcomes

Our meta-analysis uncovered significant correlations between heart rate-derived TRIMP measures and improvements in physiological markers of endurance performance, specifically VO_2max_ and V_LT_. Additionally, time spent performing at high-intensity HR showed significant correlations with improvements in both V_LT_ and V_OBLA_. These findings suggest that higher HR is consistently associated with greater improvements in common physiological endurance indicators.

#### Physiological Outcomes

Research across various included studies reported a consistent trend with TRIMP being frequently correlated with improvements in endurance performance measured as VO_2max_, running economy, Yo-Yo IRT performance, and markers at specified blood lactate concentrations (2 mmol·L^− 1^ and 4 mmol·L^− 1^). Studies examining elite athletes—including field hockey ( [58]), soccer ( [73, 74]), hurling [48], and rugby union players ( [53, 75])—noted significant correlations between TRIMP variables and positive changes in endurance performance markers. Although the reported correlations varied in magnitude across studies, most findings indicate that TRIMP variables are valuable indicators for subsequent endurance adaptations.

The significant correlation between TRIMP and improvements in endurance performance surrounding different training periods can be theoretically explained by underlying cardiovascular stress promoting aerobic adaptations [76]. In this regard, TRIMP quantifies training load by integrating HR and exercise duration, capturing the physiological strain imposed on the cardiovascular system during training [77]. Consequently, the cardiovascular stress detected with TRIMP variables may align with the progressive overload achieved, whereby repeated exposures to an appropriate stimulus likely promote adaptations in aerobic functional pathways such as oxygen transport, lactate clearance, and energy efficiency [78], which in turn may improve key endurance performance markers (e.g., VO_2max_, V_LT_).

Our meta-correlation also revealed that time spent working at high-intensity HR showed a large correlation with gains in both V_LT_ and V_OBLA_. On this note, Castagna et al. [79] and Kalapotharakos et al. [80] emphasize the critical role of training intensity in fostering endurance adaptations. Castagna et al. [79] found a significant correlation between time spent working at HR >90% of maximal HR and improvements in velocity at blood lactate concentrations of 2 mmol·L^− 1^ and 4 mmol·L^− 1^, noting however that players typically spent around two-thirds of their training time at low intensities. Similarly, Kalapotharakos et al. [80] reported that the time spent working at HR within 90–100% of maximal HR correlated significantly with performance in the interval shuttle run test, reinforcing the necessity of high-intensity training for enhancing aerobic performance. While this trend was previously observed in systematic synthesis of the literature by Fox et al. [12] in 2018, the meta-correlation we performed further validates these findings by integrating diverse contexts to provide an objective outcome. It underscores the importance of not only the time spent in high-intensity HR zones but also TRIMP variables, which reflect the total training volume accumulated. Both factors are strongly correlated with the ability to promote endurance adaptations in team sports athletes. For example, Malone and Collins [48] showed in elite hurling players that allocating ~ 20–25% of training time above 80–90% HR_max_, alongside progressive increases in overall training load (TRIMP), was strongly associated with improvements in VO₂_max_, V_LT_, and V_OBLA_ across a 12-week pre-season. This shows how a balanced distribution of high-intensity stimuli and sufficient accumulated load can be practically implemented to optimize aerobic adaptations.

#### Performance Outcomes

On the other hand, no significant correlations were observed between sRPE and adaptations in most endurance measures, with the exception of V_LT_. Furthermore, the individual studies showed considerable heterogeneity in their observations. Across multiple studies in rugby union and league [51, 53] and soccer [81, 82], a consistent trend emerged with sRPE exhibiting weak associations with changes in endurance measures such as VO_2max_ and Yo-Yo IRT performance. These findings suggest that sRPE may not be a useful indicator to gauge potential physiological adaptations in team sports.

While sRPE offers practical advantages due to its simplicity and non-invasive nature, its subjective nature may limit its representation of true physiological strain in team sport training contexts, highlighting that it captures only one aspect of the broader training load profile [83]. Aerobic performance improvements may rely heavily on specific, quantifiable stimuli to the cardiovascular and metabolic systems, and HR-based measures may better capture these physiological responses in a more direct manner than perceptually-based RPE measurements for an entire session, which may explain the inconsistency in correlations between sRPE and adaptations in endurance performance [76]. Moreover, methodological inconsistencies further undermine the utility of sRPE. These include the absence of a standardized definition of perceived exertion, the inappropriate use of non-validated scales, and the frequent omission of adequate participant familiarization procedures—all of which can compromise both research findings and applied practice [84, 85]. To enhance the value of RPE, researchers and practitioners must adopt rigorous procedures for scale selection, definition, and familiarization; otherwise, the continued collection of poorly standardized RPE data risks wasting time and resources compared with more robust measures such as HR or blood lactate. Ultimately, our results suggest that although sRPE may be useful for certain functions, such as guiding day-to-day training decisions, its effectiveness to determine long-term aerobic gains may be questionable when compared to more objective HR-based monitoring methods.

Additionally, using accumulated total distance covered during sessions to explain adaptations in endurance performance indicators appears limited based on our findings. For example, in soccer, Clemente et al. [86] found only small and unclear correlations between total accumulated distance covered and changes in VO_2max_. Similarly, another study [82] found unclear correlations between mean distances covered performing at high-speed (>17 km/h) or very high-speed (>21 km/h) running and changes in maximal aerobic speed. However, the same study [82] observed significant and large correlations between the mean weekly accumulated distance performing at velocities above the maximal aerobic speed threshold and improvements in maximal aerobic speed. Likewise, Papadakis et al. [75] revealed that high-speed running had moderate correlations with changes in VO_2max_, as well as velocities at blood lactate concentrations of 2 mmol·L^− 1^ and 4 mmol·L^− 1^. These collective observations suggest that total distance, a general measure encompassing all movement intensities, may not adequately capture aerobic performance adaptations. Instead, more specific measures encompassing loads at high intensities might yield greater associations to indicate potential for endurance performance gains. However, in contrast to these findings, Taylor et al. [53] reported a large negative relationship between very high-speed running distance (>18 km∙h⁻¹) and changes in VO₂_max_ among academy rugby union players. Because this analysis was conducted over a short, 6-week in-season period, the accumulation of very high-intensity running alongside congested competitive demands may have restricted recovery and blunted positive aerobic adaptations. This highlights potential methodological and contextual limitations of the research, rather than providing definitive evidence that high-speed running itself diminishes endurance fitness. This finding is important to note as improvements in endurance with increased load using identified measures likely sit on a continuum with a limit beyond which maladaptive responses may occur, corresponding with declines in performance [87, 88]. It should be noted that the number of studies specifically exploring distance-based measures was insufficient for meta-correlations to be determined, meaning such analyses are needed as more research becomes available in this area.

### Adaptations in Strength and power, and Speed Performance Outcomes

The availability of data pertaining to the relationships between training load measures and adaptations in strength, power, and speed is more limited compared to the data for endurance variables. However, the data that allowed for the establishment of meta-correlations revealed that sRPE had no meaningful correlations with changes in CMJ performance.

Research examining sRPE in soccer players revealed a consistent trend with trivial or unclear correlations with changes in CMJ performance as revealed by Gil-rey et al. [60] and Arcos et al. [89]. These collective findings suggest that as players accomplish greater perceptual loads, which is a function of perceived intensity and duration, they do not necessarily experience notable improvements in jump performance measures. This pattern indicates that sRPE may not be an effective marker to anticipate potential improvements in power-related performance outcomes in soccer, highlighting a potential limitation in its utility. However, emerging evidence suggests that differential RPE (dRPE), which separates ratings of respiratory (sRPE-B) and muscular exertion (sRPE-L), may offer greater sensitivity. For example, McLaren et al. [90] found that players with higher weekly sRPE-B training loads during pre-season were more likely to show improvements in CMJ performance, whereas global sRPE-TL showed no such relationship. This indicates that dRPE could provide a more nuanced understanding of load–adaptation pathways and warrants consideration in both future research and applied monitoring.

The heterogeneity observed in the results across studies may stem from the fact that sRPE serves predominantly as a volume-related measure, providing a broad overview of the psychophysiological training load [85]. In contrast, it lacks the specificity required to capture stimuli that may underpin the nuanced neuromuscular adaptations essential for performance in jumping tests. These tests and physical qualities primarily depend on enhancements in neural stimulation and mechanical work [91] rather than merely reflecting the overall psychophysiological demands encountered. Consequently, the general nature of sRPE may compromise the strength of its association with specific adaptations required for improvements in jumping performance. In this regard, more specific mechanical measures, such as those derived from IMUs, might be of greater relevance to infer potential power-related improvements during jumping tests. At the same time, the emerging use of dRPE [90] offers another avenue to address this limitation, as separating respiratory and muscular exertion may provide a more targeted perceptual perspective to complement objective mechanical measures. Future research should therefore explore dRPE alongside mechanical monitoring tools to better capture neuromuscular adaptation pathways.

The diversity of results concerning correlations between total distance covered and changes in strength and power measures is noteworthy. Across studies in rugby union [52] and soccer [75], a prevailing trend was that both acute and chronic loads may negatively impact on performance metrics such as 1-RM bench press and CMJ height. In rugby union players [52], a small negative correlation was found between acute heavy impacts and 1-RM bench press performance, while chronic workload (workload accumulated) negatively correlated with both 1-RM bench pull and CMJ height performance. Similarly, in soccer players [75], higher total distances were small and negatively correlated with changes in squat jump height and CMJ height performance compared to those with lower distances. Notably, high-speed running also showed a negative association with changes in squat jump performance. These findings suggest that excessive external loads could hinder performance adaptations in strength and jump-related tests across sports, although no meta-correlation could be performed for objective quantification of this association.

Finally, regarding the correlations between training load and changes in speed, the meta-correlation conducted did not identify significant correlations between sRPE and changes in linear speed tests across *≤* 10 m. In fact, findings across studies even indicated negative associations between sRPE and changes in linear sprint times, although many studies reported trivial or small magnitudes [49, 92]. Likewise, research reporting on the relationship between distance covered at high intensities and changes in linear sprint performance observed negative, small relationships [82]. Consequently, this collective research suggests that internal perceptual and external intensity-oriented training load measures may offer limited use in anticipating potential improvements in linear speed, but in fact promote some deterioration if administered at certain levels.

### Limitations and Future Research

Our systematic review and meta-analysis aimed to provide a current synthesis of evidence regarding the correlations between training load and adaptations in physical performance. Unlike previous reviews in this area [12, 13], which primarily summarized individual studies without synthesizing data for meta-analysis, our goal was to consolidate and analyze the available data. However, this process was challenging and restricted with inherent limitations, largely due to the diversity of studies in this area.

One significant limitation encountered was the uneven distribution of data across time. Training load monitoring has been studied for two decades [3], but research reporting external load is mostly concentrated within the last decade. This confines the scope of the research to the tools and methods available for training load monitoring. In team sports, for example, external load is typically measured using GPS, which has prohibited use within indoor environments. This restriction introduces a bias, as most studies reporting external load have focused on outdoor sports like soccer and rugby union or league, with significantly less research conducted in other sports. The specific demands of different sports may influence the magnitude and trends of correlations observed in performance measures. Indoor sports typically emphasize acceleration, deceleration, changes of direction, and jumping, while outdoor sports often involve greater spaces that require players to rely more on accumulated distance and sustained high-intensity running.

Furthermore, the wide range of measures used to monitor both internal and external load, as well as the diversity of physical performance outcomes, posed a considerable challenge in organizing the data into domains suitable for meta-analysis. As a result, the selection and grouping of measures for analysis requires consideration when interpreting our findings. Based on our review, it can be argued that TRIMP and the time spent working at high-intensity HR are critical indicators for monitoring changes in endurance performance. However, it was also observed that various common training load measures may not be useful in anticipating potential adaptations in strength, power, and speed within team sports.

It is also important for future studies to incorporate larger datasets, as this could enable the use of advanced techniques such as machine learning. Such approaches could help identify different types of responders to training loads, providing valuable insights into which training load measures are most relevant based on factors such as playing position, trainability, age, or even the characteristics of individual athletes. Such advancements would represent a significant step towards more individualized evidence to inform the development of training programs designed for the specific needs of players, using data to target desired adaptations more precisely.

In addition, future research should seek to better understand how exposure to different levels of external load metrics (i.e., basic locomotor outputs such as total distance, metabolic power or individualized speed thresholds, or mechanical/acceleration–deceleration/jump demands) influences both physiological markers (e.g., VO₂_max_, lactate thresholds) and performance outcomes (e.g., sprint speed, repeated-sprint ability, jumping). Clarifying the effects of these distinct load categories could help determine which dimensions of external load are most predictive of specific adaptations in team sport athletes.

Finally, future research should consider not only the quantity of training load but also its quality. For example, the same training load could be applied across different exercises; however, the specific type of exercise and its focus are crucial factors that should be documented. This information could be provided in the form of a supplementary file accompanying individual studies so that future syntheses of the literature can account for it. Such an approach would enhance the design of studies by adding greater detail and context to the data.

## Conclusions

TRIMP and the time spent performing at high-intensity HR were found to be the most relevant training load measures, being strongly (moderate-to-large correlations) associated with endurance performance adaptations in team sport athletes. Conversely, while sRPE was the primary training load measure examined across studies, its associations appeared limited—showing a small positive correlation with V_LT_ as an endurance marker, and a small negative correlation with linear sprint performance > 10 m that attenuated to trivial in sensitivity analysis—while generally demonstrating no significant correlations with adaptations related to strength or jump-related power outcomes.

## Supplementary Information

Below is the link to the electronic supplementary material.

Supplementary Material 1

Supplementary Material 2

Supplementary Material 3

Supplementary Material 4

Supplementary Material 5

## Data Availability

All data are available by request to the corresponding author.

## Abbreviations

CMJ: Countermovement jump
GRADE: Grading of Recommendations Assessment, Development and Evaluation methodology
HR: Heart rate
sRPE: Session rating of perceived exertion
TRIMP: Training impulse
V_LT_: Velocity at a blood lactate concentration of 2 mmol.L^− 1^
V_OBLA_: Velocity at a blood lactate concentration of 4 mmol.L^− 1^
VO_2max_: Maximal oxygen uptake
