# Supplementary material for: The Correlations Between Training Load Parameters and Physical Performance Adaptations in Team Sports: A Systematic Review and Meta-analysis
Source: Sports Med Open. 2025 Dec 11;11:156. doi: 10.1186/s40798-025-00952-4 (PMC12698923; doi:10.1186/s40798-025-00952-4)
Supplement: Supplementary file 1 — Supplementary Material 1 [file 40798_2025_952_MOESM1_ESM.docx]

| **Supplementary material 2.** Correlation between indicators of endurance (aerobic performance and lactate) and load. | | | |
| --- | --- | --- | --- |
| **Study** | **Performance measure** | **Training load variable** | **Correlation coefficient (*r*)** |
| **Aerobic endurance measures** | | | |
| Stagno et al. [58] | VO_2max_ | Mean weekly TRIMP modified | 0.80 |
|  | VO_2max_ | Mean weekly time spent in high intensity activity^1^ | 0.62 |
| Castagna et al. [94] | VO_2max_ | Time spent in high intensity activity^2^ | 0.65 |
|  | Yo-yo IR1 | Time spent in high intensity activity^2^ | 0.66 |
| Manzi et al. [74] | VO_2max_ | Mean weekly iTRIMP | 0.77 |
|  | Yo-yo IR1 | Mean weekly iTRIMP | 0.69 |
|  | VO_2VT_ | Mean weekly iTRIMP | 0.78 |
| Gil-Rey et al. [60] | Time to exhaustion | Total accumulated sRPEresp | 0.71 |
|  | Time to exhaustion | Total accumulated sRPEmus | 0.69 |
|  | Time to exhaustion | Training and match volume | 0.67 |
| Malone et al. [48] | VO_2max_ | Weekly iTRIMP | 0.77 |
|  | Yo-yo IR1 | Weekly iTRIMP | 0.79 |
|  | Yo-yo IR2 | Weekly iTRIMP | 0.92 |
| Campos-Vazquez et al. [96] | V_IFT_ | sRPE | 0.70 |
|  | V_IFT_ | sumRPE | 0.75 |
|  | V_IFT_ | Edwards’_RES_ | 0.25 |
|  | V_IFT_ | Training and match volume | 0.72 |
| Dobbin et al. [50] | Yo-yo IR1 | sRPE_RT_ | 0.04 |
|  | Yo-yo IR1 | sRPE_COND_ | 0.01 |
|  | Yo-yo IR1 | sRPE_SK_ | 0.11 |
|  | Yo-yo IR1 | Total sRPE | 0.07 |
| Ferioli et al. [56] | Yo-yo IR1 | Weekly sRPE | 0.18 |
|  | Yo-yo IR1 | Training volume | 0.10 |
| Fitzpatrick et al. [82] | MAS | sRPE | -0.22 |
|  | MAS | eTRIMP | -0.21 |
|  | MAS | Total distance | 0.26 |
|  | MAS | Acceleration and deceleration distance > 2 m^.^s^-2^ | 0.20 |
|  | MAS | HSD | 0.22 |
|  | MAS | VHSD | -0.07 |
|  | MAS | Meters above MAS | 0.50 |
|  | MAS | Time above MAS | 0.77 |
|  | MAS | Meters above 30% MAS | 0.20 |
|  | MAS | Time above 30% MAS | 0.62 |
| Figueiredo et al. [61] | Yo-yo IR1 | U15: Weekly sRPE | -0.50 |
|  | Yo-yo IR1 | U15: Monotony | -0.87 |
|  | Yo-yo IR1 | U15: Strain | -0.37 |
|  | Yo-yo IR1 | U17: Weekly sRPE | -0.18 |
|  | Yo-yo IR1 | U17: Monotony | -0.68 |
|  | Yo-yo IR1 | U17: Strain | -0.77 |
| Taylor et al. [53] | VO_2max_ | sRPE | 0.35 |
|  | VO_2max_ | iTRIMP | 0.74 |
|  | VO_2max_ | luTRIMP | 0.55 |
|  | VO_2max_ | eTRIMP | 0.63 |
|  | VO_2max_ | bTRIMP | 0.88 |
|  | VO_2max_ | Total distance | -0.51 |
|  | VO_2max_ | Player load | -0.24 |
|  | VO_2max_ | iHSD | -0.26 |
|  | VO_2max_ | 15HSD | -0.19 |
|  | V_VO2max_ | 18HSD | -0.63 |
|  | V_VO2max_ | sRPE | 0.37 |
|  | V_VO2max_ | iTRIMP | 0.39 |
|  | V_VO2max_ | luTRIMP | 0.70 |
|  | V_VO2max_ | eTRIMP | 0.14 |
|  | V_VO2max_ | bTRIMP | 0.51 |
|  | V_VO2max_ | Total distance | -0.01 |
|  | V_VO2max_ | Player load | 0.17 |
|  | V_VO2max_ | iHSD | 0.34 |
|  | V_VO2max_ | 15HSD | 0.32 |
|  | V_VO2max_ | 18HSD | -0.16 |
| Rabbani et al. [97] | V_IFT_ | Time spent Z5 TRIMP: > 90% maximal heart rate | 0.72 |
|  | V_IFT_ | bTRIMP | 0.51 |
|  | V_IFT_ | eTRIMP | 0.54 |
|  | V_IFT_ | New body load | 0.54 |
|  | V_IFT_ | HIR | 0.23 |
|  | V_IFT_ | VHIR | 0.06 |
|  | V_IFT_ | Training volume | 0.26 |
|  | V_IFT_ | Total distance | 0.33 |
| Clemente et al. [81] | VO_2max_ | Srpe | 0.21 |
|  | VO_2max_ | Monotony | 0.23 |
|  | VO_2max_ | Strain | -0.14 |
|  | MAS | sRPE | 0.22 |
|  | MAS | Monotony | -0.04 |
|  | MAS | Strain | 0.28 |
| Clemente et al. [86] | VO_2max_ | Training volume | -0.56 |
|  | VO_2max_ | Total distance | -0.15 |
|  | VO_2max_ | Sprint distance | -0.46 |
|  | VO_2max_ | Sum of accelerations | 0.58 |
| Daniels et al. [51] | Yo-yo IR1 | Weekly sRPE | -0.21 |
| Saidi et al. [98] | Yo-yo IR1 | Weekly sRPE | 0.14 |
|  | Yo-yo IR1 | Monotony | -0.01 |
|  | Yo-yo IR1 | Strain | 0.12 |
| Azcárate et al. [99] | VO_2max_ | match sRPEmus | 0.56 |
|  | VO_2max_ | training sRPEresp | -0.53 |
|  | MAS | match sRPEmus | 0.56 |
|  | MAS | training sRPEresp | -0.53 |
| Malone et al. [47]^3^ | Yo-yo IR1 | Time spent at heart rate high intensity | 0.65 |
| Malone et al. [49] | VO_2max_ | sRPE | 0.13 |
|  | VO_2max_ | iTRIMP | 0.68 |
|  | VO_2max_ | luTRIMP | 0.28 |
|  | VO_2max_ | eTRIMP | 0.19 |
|  | VO_2max_ | bTRIMP | 0.28 |
|  | VO_2max_ | gTRIMP | 0.19 |
|  | MAS | sRPE | 0.46 |
|  | MAS | iTRIMP | 0.56 |
|  | MAS | luTRIMP | 0.47 |
|  | MAS | eTRIMP | 0.37 |
|  | MAS | bTRIMP | 0.66 |
|  | MAS | gTRIMP | 0.46 |
|  | V_VO2max_ | sRPE | 0.41 |
|  | V_VO2max_ | iTRIMP | 0.66 |
|  | V_VO2max_ | luTRIMP | 0.41 |
|  | V_VO2max_ | eTRIMP | 0.38 |
|  | V_VO2max_ | bTRIMP | 0.66 |
|  | V_VO2max_ | gTRIMP | 0.40 |
|  | Yo-yo IR1 | sRPE | 0.12 |
|  | Yo-yo IR1 | iTRIMP | 0.69 |
|  | Yo-yo IR1 | luTRIMP | 0.11 |
|  | Yo-yo IR1 | eTRIMP | 0.15 |
|  | Yo-yo IR1 | bTRIMP | 0.65 |
|  | Yo-yo IR1 | gTRIMP | 0.21 |
|  | Yo-yo IR2 | sRPE | 0.11 |
|  | Yo-yo IR2 | iTRIMP | 0.59 |
|  | Yo-yo IR2 | luTRIMP | 0.21 |
|  | Yo-yo IR2 | eTRIMP | 0.21 |
|  | Yo-yo IR2 | bTRIMP | 0.46 |
|  | Yo-yo IR2 | gTRIMP | 0.33 |
| Papadakis et al. [75] | VO_2max_ | bTRIMP | 0.46 |
|  | VO_2max_ | Total distance | 0.14 |
|  | VO_2max_ | HSD | 0.45 |
|  | VO_2max_ | SD | 0.58 |
| Ellis et al. [73] | MAS | sRPE | 0.37 |
|  | MAS | iTRIMP | 0.37 |
|  | MAS | bTRIMP | 0.03 |
|  | MAS | luTRIMP | 0.26 |
|  | MAS | eTRIMP | 0.08 |
|  | MAS | Total distance | 0.34 |
|  | MAS | HSD | 0.11 |
|  | MAS | VHSD | -0.06 |
|  | MAS | iHSD | 0.27 |
|  | MAS | Maximal SD | 0.10 |
|  | MAS | Player load | 0.56 |
| Figueiredo et al. [71] | Yo-yo IR1 | Weekly sRPE | -0.69 |
|  | Yo-yo IR1 | eTRIMP | -0.50 |
| Kalapotharakos et al. [80] ^4^ | Shuttle run | Time at high-intensity heart rate zone | 0.59 |
| Younesi et al. [100] | V_IFT_ | sRPE | 0.08 |
|  | V_IFT_ | sRPEresp | 0.03 |
|  | V_IFT_ | sRPEmusc | 0.08 |
|  | V_IFT_ | TRIMP | 0.42 |
|  | V_Vavemal_ | sRPE | 0.51 |
|  | V_Vavemal_ | sRPEresp | 0.53 |
|  | V_Vavemal_ | sRPEmusc | 0.36 |
|  | V_Vavemal_ | TRIMP | 0.01 |
| Rabbani et al. [102] | HR_EXERCISE_ | Total minutes | -0.71 |
|  | HR_EXERCISE_ | Total distance | -0.65 |
|  | HR_EXERCISE_ | Mechanical work | -0.42 |
|  | HR_EXERCISE_ | High metabolic load distance | -0.38 |
|  | HR_EXERCISE_ | eTRIMP | -0.35 |
| Xiong et al. [103] | Yo-yo IR1 | sRPE | 0.67 |
|  | Yo-yo IR1 | eTRIMP | 0.65 |
|  | Yo-yo IR1 | Total distance | -0.10 |
|  | Yo-yo IR1 | 14-19VHSR (m) | -0.01 |
|  | Yo-yo IR1 | 20VHSR (m) | -0.05 |
| Perrotta et al. [105] | Yo-yo IR1 | Training load Polar | 0.76 |
|  | Yo-yo IR1 | eTRIMP | 0.63 |
|  | Yo-yo IR1 | Total distance | 0.43 |
|  | Yo-yo IR1 | Sprint number | 0.15 |
|  | Yo-yo IR1 | Acceleration number | -0.48 |
|  | Yo-yo IR1 | Deceleration number | -0.26 |
| Savolainen et al. [106] | MAS | eTRIMP: total duration | 0.50 |
|  | MAS | eTRIMP: total distance | 0.40 |
|  | MAS | eTRIMP: 13LIRD | 0.44 |
|  | MAS | eTRIMP: 13-19HIRD | 0.44 |
|  | MAS | eTRIMP: 19VHIRD | 0.41 |
|  | MAS | eTRIMP:low acceleration | 0.39 |
|  | MAS | eTRIMP: moderate acceleration | 0.42 |
|  | MAS | eTRIMP: high aceleration | 0.51 |
|  | MAS | HR_MEAN_: total distance | 0.59 |
|  | MAS | HR_MEAN_:13LIRD | 0.50 |
|  | MAS | HR_MEAN_: 13-19HIRD | 0.63 |
|  | MAS | HR_MEAN_: 19VHIRD | 0.45 |
|  | MAS | HR_MEAN_: low acceleration | 0.41 |
|  | MAS | HR_MEAN_: moderate acceleration | 0.55 |
|  | MAS | HR_MEAN_: high aceleration | 0.57 |
| **Lactate outputs** | | | |
| **Study** | **Performance measure** | **Training load variable** | **Correlation coefficient (*r*)** |
| Gorostiaga et al. [54]^5^ | Velocity at blood lactate concentration of 3 mmol^.^L^-1^ | Endurance and ball exercise training time at high intensity | 0.68 |
| Stagno et al. [58] | V_OBLA_ | Mean weekly TRIMP modified | 0.71 |
|  | V_OBLA_ | Mean weekly time spent in high intensity activity^1^ | 0.62 |
| Castagna et al. [79] | V_LT_ | Time spent in high intensity activity^2^ | 0.84 |
|  | V_OBLA_ | Time spent in high intensity activity^2^ | 0.65 |
| Akubat et al. [93] | V_LT_ | Mean weekly sRPE | 0.13 |
|  | V_LT_ | bTRIMP | 0.28 |
|  | V_LT_ | iTRIMP | 0.67 |
|  | V_LT_ | Team TRIMP | 0.20 |
|  | V_OBLA_ | Mean weekly sRPE | 0.40 |
|  | V_OBLA_ | bTRIMP | 0.43 |
|  | V_OBLA_ | iTRIMP | 0.33 |
|  | V_OBLA_ | Team TRIMP | 0.28 |
|  | HR_LT_ | Mean weekly sRPE | 0.20 |
|  | HR_LT_ | bTRIMP | 0.21 |
|  | HR_LT_ | iTRIMP | 0.17 |
|  | HR_LT_ | Team TRIMP | 0.28 |
|  | HR_OBLA_ | Mean weekly sRPE | 0.15 |
|  | HR_OBLA_ | bTRIMP | -0.21 |
|  | HR_OBLA_ | iTRIMP | -0.25 |
|  | HR_OBLA_ | Team TRIMP | -0.49 |
| Castagna et al. [94] | V_LT_ | Time spent in high intensity activity^2^ | 0.78 |
|  | V_OBLA_ | Time spent in high intensity activity^2^ | 0.69 |
| Manzi et al. [74 | V_OBLA_ | Mean weekly iTRIMP | 0.64 |
| Arcos et al. [92] | Velocity at blood lactate concentration of 3 mmol^.^L^-1^ | sRPEresp | -0.30 |
|  | Velocity at blood lactate concentration of 3 mmol^.^L^-1^ | sRPEmusc | -0.45 |
|  | Velocity at blood lactate concentration of 3 mmol^.^L^-1^ | sumRPEresp | -0.17 |
|  | Velocity at blood lactate concentration of 3 mmol^.^L^-1^ | sumRPEmusc | -0.33 |
|  | Velocity at blood lactate concentration of 3 mmol^.^L^-1^ | Training volume | 0.31 |
|  | Blood lactate at 12 km^.^h^-1^ | sRPEresp | 0.01 |
|  | Blood lactate at 12 km^.^h^-1^ | sRPEmusc | -0.29 |
|  | Blood lactate at 12 km^.^h^-1^ | sumRPEresp | 0.13 |
|  | Blood lactate at 12 km^.^h^-1^ | sumRPEmusc | -0.20 |
|  | Blood lactate at 12 km^.^h^-1^ | Training volume | 0.21 |
|  | Blood lactate at 13 km^.^h^-1^ | sRPEresp | -0.36 |
|  | Blood lactate at 13 km^.^h^-1^ | sRPEmusc | -0.57 |
|  | Blood lactate at 13 km^.^h^-1^ | sumRPEresp | -0.28 |
|  | Blood lactate at 13 km^.^h^-1^ | sumRPEmusc | -0.48 |
|  | Blood lactate at 13 km^.^h^-1^ | Training volume | 0.37 |
| Malone et al. [48] | V_LT_ | Weekly iTRIMP | 0.64 |
| Arcos et al. [95] | Velocity at blood lactate concentration of 3 mmol^.^L^-1^ | sRPEresp | -0.57 |
|  | Velocity at blood lactate concentration of 3 mmol^.^L^-1^ | sRPEmusc | -0.47 |
|  | Velocity at blood lactate concentration of 3 mmol^.^L^-1^ | sumRPEresp | -0.43 |
|  | Velocity at blood lactate concentration of 3 mmol^.^L^-1^ | sumRPEmusc | -0.26 |
|  | Velocity at blood lactate concentration of 3 mmol^.^L^-1^ | Training volume | 0.57 |
|  | Blood lactate at 12 km^.^h^-1^ | sRPEresp | -0.34 |
|  | Blood lactate at 12 km^.^h^-1^ | sRPEmusc | -0.14 |
|  | Blood lactate at 12 km^.^h^-1^ | sumRPEresp | -0.16 |
|  | Blood lactate at 12 km^.^h^-1^ | sumRPEmusc | 0.10 |
|  | Blood lactate at 12 km^.^h^-1^ | Training volume | 0.62 |
|  | Blood lactate at 13 km^.^h^-1^ | sRPEresp | -0.61 |
|  | Blood lactate at 13 km^.^h^-1^ | sRPEmusc | -0.55 |
|  | Blood lactate at 13 km^.^h^-1^ | sumRPEresp | -0.49 |
|  | Blood lactate at 13 km^.^h^-1^ | sumRPEmusc | -0.34 |
|  | Blood lactate at 13 km^.^h^-1^ | Training volume | 0.61 |
| Ferioli et al. [56] | Mognoni’s_LA_ | Weekly sRPE | -0.14 |
|  | Mognoni’s_LA_ | Training volume | 0.04 |
|  | HIT_LA_ | Weekly sRPE | -0.48 |
|  | HIT_LA_ | Training volume | 0.32 |
| Taylor et al. [53] | V_OBLA_ | sRPE | 0.26 |
|  | V_OBLA_ | iTRIMP | 0.20 |
|  | V_OBLA_ | luTRIMP | 0.14 |
|  | V_OBLA_ | eTRIMP | 0.52 |
|  | V_OBLA_ | bTRIMP | 0.46 |
|  | V_OBLA_ | Total distance | -0.31 |
|  | V_OBLA_ | Player load | -0.47 |
|  | V_OBLA_ | iHSD | -0.27 |
|  | V_OBLA_ | 15HSD | -0.25 |
|  | V_OBLA_ | 18HSD | -0.16 |
|  | V_LT_ | sRPE | 0.33 |
|  | V_LT_ | iTRIMP | 0.47 |
|  | V_LT_ | luTRIMP | 0.45 |
|  | V_LT_ | eTRIMP | 0.33 |
|  | V_LT_ | bTRIMP | 0.56 |
|  | V_LT_ | Total distance | -0.21 |
|  | V_LT_ | Player load | -0.03 |
|  | V_LT_ | iHSD | 0.12 |
|  | V_LT_ | 15HSD | -0.06 |
|  | V_LT_ | 18HSD | -0.43 |
| Malone et al. [47]^3^ | V_OBLA_ | Time spent at heart rate high intensity | 0.77 |
|  | V_LT_ | Time spent at heart rate high intensity | 0.80 |
| Malone et al. [49] | V_OBLA_ | sRPE | 0.13 |
|  | V_OBLA_ | iTRIMP | 0.68 |
|  | V_OBLA_ | luTRIMP | 0.28 |
|  | V_OBLA_ | eTRIMP | 0.19 |
|  | V_OBLA_ | bTRIMP | 0.28 |
|  | V_OBLA_ | gTRIMP | 0.19 |
|  | V_LT_ | sRPE | 0.40 |
|  | V_LT_ | iTRIMP | 0.78 |
|  | V_LT_ | luTRIMP | 0.21 |
|  | V_LT_ | eTRIMP | 0.38 |
|  | V_LT_ | bTRIMP | 0.44 |
|  | V_LT_ | gTRIMP | 0.28 |
| Papadakis et al. [75] | V_OBLA_ | bTRIMP | 0.31 |
|  | V_OBLA_ | Total distance | 0.42 |
|  | V_OBLA_ | HSD | 0.39 |
|  | V_OBLA_ | SD | 0.36 |
|  | V_LT_ | bTRIMP | 0.06 |
|  | V_LT_ | Total distance | 0.60 |
|  | V_LT_ | HSD | 0.45 |
|  | V_LT_ | SD | 0.26 |
| Ellis et al. [73] | V_OBLA_ | sRPE | -0.16 |
|  | V_OBLA_ | iTRIMP | 0.88 |
|  | V_OBLA_ | bTRIMP | 0.18 |
|  | V_OBLA_ | luTRIMP | 0.82 |
|  | V_OBLA_ | eTRIMP | 0.01 |
|  | V_OBLA_ | Total distance | -0.11 |
|  | V_OBLA_ | HSD | -0.45 |
|  | V_OBLA_ | VHSD | -0.33 |
|  | V_OBLA_ | iHSD | -0.12 |
|  | V_OBLA_ | Maximal SD | -0.15 |
|  | V_OBLA_ | Player load | 0.51 |
|  | V_LT_ | sRPE | -0.17 |
|  | V_LT_ | iTRIMP | 0.93 |
|  | V_LT_ | bTRIMP | 0.33 |
|  | V_LT_ | luTRIMP | 0.75 |
|  | V_LT_ | eTRIMP | 0.17 |
|  | V_LT_ | Total distance | -0.14 |
|  | V_LT_ | HSD | -0.45 |
|  | V_LT_ | VHSD | -0.25 |
|  | V_LT_ | iHSD | 0.01 |
|  | V_LT_ | Maximal SD | -0.22 |
|  | V_LT_ | Player load | 0.49 |
| Notes: ^1^High-intensity activity corresponds to zones 4 (86-92%) and 5 (93-100%) of maximal heart rate. ^2^Heart rate > 4 mmol^.^L^-1^. ^3^Heart rate was categorized as low intensity (≤ HR at 2 mmol·L^-1^), medium intensity (between HR at 2 and 4 mmol·L^-1^), and high intensity (≥ HR at 4 mmol·L^-1^). ^4^High intensity heart rate zone corresponds to 90-100% of maximal heart rate. ^5^The correlations were performed for specific periods of training. Abbreviations outcomes: VO_2max_ (maximal oxygen uptake), Yo-yo IR1 (Yoyo Intermittent Recovery Level 1), VO_2VT_ (ventilatory threshold at maximal oxygen uptake), Yo-yo IR2 (Yoyo Intermittent Recovery Level 2), V_IFT_ (final velocity 30-15 Intermittent Fitness Test), MAS (maximal aerobic speed), V_VO2max_ (velocity at VO_2max_), V_Vavemal_ (final velocity reached in last stage), V_OBLA_ (speed at 4 mmol^.^L^-1^), V_LT_ (speed at 2 mmol^.^L^-1^), HR_LT_ (heart rate at 2 mmol^.^L^-1^), HR_OBLA_ (heart rate at 4 mmol^.^L^-1^), Mognoni’s_LA_ (Mognoni’s continuous test blood lactate concentration), HIT_LA_ (high-intensity intermittent running test blood lactate). Abbreviations load indicators: TRIMP (training impulse); iTRIMP (individualized training impulse), sRPEresp (session rating perceived exertion respiratory), sRPEmus (session rating perceived exertion local-muscular), sRPE (session rating perceived exertion), Edwards’_RES_ (Edwards’ reserve), sRPE_RT_ (session rating perceived exertion resistance training), sRPE_COND_ (session rating perceived exertion conditioning), sRPE_SK_ (session rating of perceived exertion skills), eTRIMP (Edward’s training impulse), HSD (high-speed distance),VHSD (very high speed distance), luTRIMP (Lucia’s training impulse), bTRIMP (Bannister’s training impulse), iHSD (individualized high speed distance), 15HSD (high speed distance > 15 km^.^h^-1^), 18HSD (high speed distance > 18 km^.^h^-1^), time spent Z5 TRIMP (time spent > 90% maximal heart rate), HIR (distance covered > 14.4 km^.^h^-1^), VHIR (distance covered > 19.8 km^.^h^-1^), gTRIMP (Stagno individualized training impulse), SD (sprint distance); 14-19HSRD (high-speed running distance 14-19 km^.^h^-1^), 20VHSRD (very high-intensity running distance covered >20.0 km^.^h^-1^); 13LIRD (low-intensity running distance > 13 km^.^h^-1^); 13-19HIRD (high-intensity running distance 13-19 km^.^h^-1^), 19VHIRD (very high-intensity running distance > 19 km^.^h^-1^). | | | |
