# Supplementary material for: The Correlations Between Training Load Parameters and Physical Performance Adaptations in Team Sports: A Systematic Review and Meta-analysis
Source: Sports Med Open. 2025 Dec 11;11:156. doi: 10.1186/s40798-025-00952-4 (PMC12698923; doi:10.1186/s40798-025-00952-4)
Supplement: Supplementary file 2 — Supplementary Material 2 [file 40798_2025_952_MOESM2_ESM.docx]

| **Supplementary material 3.** Correlations between load and power outputs. | | | |
| --- | --- | --- | --- |
| **Study** | **Outcome** | **Load indicator** | **Correlation coefficient (*r* value)** |
| **Explosive power** | | | |
| Gorostiaga et al. [54]^1^ | Standing throwing velocity | Total strength training time | 0.58 |
|  | Power at 125% of body mass half squat | Total strength training time | -0.79 |
| Granados et al. [55] | Velocity at 30% of maximal repetition bench press | Competition and training volume | 0.58 |
| Arcos et al. [95] | CMJ height | Competition and training volume | -0.52 |
| Arcos et al. [92] | CMJ height | sRPEresp | -0.44 |
|  | CMJ height | sRPEmusc | -0.20 |
|  | CMJ height | sumRPEresp | -0.43 |
|  | CMJ height | sumRPEmusc | -0.17 |
|  | CMJ height | Training volume | 0.42 |
|  | CMJ arm swing height | sRPEresp | -0.29 |
|  | CMJ arm swing height | SRPEmusc | -0.40 |
|  | CMJ arm swing height | sumRPEresp | -0.25 |
|  | CMJ arm swing height | sumRPEmusc | -0.38 |
|  | CMJ arm swing height | Training volume | 0.51 |
|  | CMJ dominant leg height | sRPEresp | -0.23 |
|  | CMJ dominant leg height | sRPEmusc | -0.54 |
|  | CMJ dominant leg height | sumRPEresp | -0.27 |
|  | CMJ dominant leg height | sumRPEmusc | -0.61 |
|  | CMJ dominant leg height | Training volume | 0.27 |
|  | CMJ non-dominant leg height | sRPEresp | -0.30 |
|  | CMJ non-dominant leg height | sRPEmusc | -0.52 |
|  | CMJ non-dominant leg height | sumRPEresp | -0.30 |
|  | CMJ non-dominant leg height | sumRPEmusc | -0.53 |
|  | CMJ non-dominant leg height | Training volume | 0.39 |
| Gil Rey et al. [60] | CMJ height | sRPEresp | -0.06 |
|  | CMJ height | sRPEmusc | -0.17 |
|  | CMJ height | Training and match volume | 0.10 |
|  | CMJ arm swing height | sRPEresp | 0.25 |
|  | CMJ arm swing height | sRPEmusc | 0.17 |
|  | CMJ arm swing height | Training and match volume | 0.23 |
| Nakamura et al. [59] | SJ height | Weekly sRPE | -0.15 |
|  | CMJ height | Weekly sRPE | -0.16 |
|  | JS | Weekly sRPE | -0.43 |
| Arcos et al. [89] | CMJ height | sRPEresp | 0.10 |
|  | CMJ height | sRPEmusc | 0.13 |
|  | CMJ height | sumRPEresp | 0.18 |
|  | CMJ height | sumRPEmusc | 0.12 |
|  | CMJ height | Training volume | 0.36 |
|  | CMJ arm swing height | sRPEresp | 0.31 |
|  | CMJ arm swing height | sRPEmusc | 0.30 |
|  | CMJ arm swing height | sumRPEresp | -0.46 |
|  | CMJ arm swing height | sumRPEmusc | 0.34 |
|  | CMJ arm swing height | Training volume | 0.32 |
| Dubois et al. [45] | Drop jump power index | Weekly sRPE | -0.40 |
|  | Drop jump power index | Total distance | -0.49 |
|  | Drop jump power index | Running speed > 13 km^.^h^-1^ | 0.27 |
|  | Drop jump power index | Distance covered at running speed > 13 km^.^h^-1^ | 0.38 |
| Dobbin et al. [52] | CMJ height | sRPE_RT_ | 0.51 |
|  | CMJ height | sRPE_COND_ | 0.19 |
|  | CMJ height | sRPE_SK_ | 0.60 |
|  | CMJ height | Total sRPE | 0.55 |
|  | Medicine ball throw distance | sRPE_RT_ | 0.40 |
|  | Medicine ball throw distance | sRPE_COND_ | 0.03 |
|  | Medicine ball throw distance | sRPE_SK_ | 0.22 |
|  | Medicine ball throw distance | Total sRPE | 0.29 |
| Daniels et al. [51] | JS | Weekly sRPE | 0.01 |
|  | Bench throw | Weekly sRPE | -0.66 |
| Saidi et al. [98] | SJ height | Weekly sRPE | -0.54 |
|  | SJ height | Monotony | 0.14 |
|  | SJ height | Strain | -0.03 |
| Papadakis et al. [75] | SJ height | bTRIMP | 0.02 |
|  | SJ height | Total distance | -0.15 |
|  | SJ height | HSD | -0.20 |
|  | SJ height | SD | -0.15 |
|  | CMJ height | bTRIMP | -0.15 |
|  | CMJ height | Total distance | -0.29 |
|  | CMJ height | HSD | -0.13 |
|  | CMJ height | SD | -0.11 |
| Ellis et al. [101] | CMJ height | sRPE | 0.48 |
|  | CMJ height | iTRIMP | 0.47 |
|  | CMJ height | luTRIMP | 0.47 |
|  | CMJ height | eTRIMP | 0.47 |
|  | CMJ height | Total distance | 0.48 |
|  | CMJ height | HSD | 0.50 |
|  | CMJ height | VHD | 0.49 |
|  | CMJ height | iHSD | 0.49 |
|  | CMJ height | Maximal SD | 0.48 |
|  | CMJ height | Player load | 0.48 |
|  | CMJ height | Distance accelerations | 0.49 |
|  | CMJ height | Distance decelerations | 0.49 |
| Xiong et al. [103] | CMJ height | sRPE | -0.05 |
|  | CMJ height | eTRIMP | -0.06 |
|  | CMJ height | Total distance | 0.20 |
|  | CMJ height | 14-19VHSR (m) | 0.08 |
|  | CMJ height | 20VHSR (m) | -0.09 |
| Perrota et al. [105] | CMJ height | Training load Polar | -0.22 |
|  | CMJ height | eTRIMP | -0.24 |
|  | CMJ height | Total distance | -0.10 |
|  | CMJ height | Sprint number | 0.17 |
|  | CMJ height | Acceleration number | 0.04 |
|  | CMJ height | Deceleration number | 0.16 |
| Savolainen et al. [106] | CMJ height | eTRIMP: total duration | -0.42 |
|  | CMJ height | eTRIMP: total distance | -0.32 |
|  | CMJ height | eTRIMP: 13LIRD | -0.32 |
|  | CMJ height | eTRIMP: 13-19HIRD | 0.39 |
|  | CMJ height | eTRIMP: 19VHIRD | 0.40 |
|  | CMJ height | eTRIMP:low acceleration | 0.38 |
|  | CMJ height | eTRIMP: moderate acceleration | 0.41 |
|  | CMJ height | eTRIMP: high aceleration | 0.50 |
|  | CMJ height | HR_MEAN_: total distance | -0.52 |
|  | CMJ height | HR_MEAN_:13LIRD | 0.49 |
|  | CMJ height | HR_MEAN_: 13-19HIRD | 0.38 |
|  | CMJ height | HR_MEAN_: 19VHIRD | 0.57 |
|  | CMJ height | HR_MEAN_: low acceleration | 0.48 |
|  | CMJ height | HR_MEAN_: moderate acceleration | 0.53 |
|  | CMJ height | HR_MEAN_: high aceleration | 0.59 |
|  |  |  |  |
| **Anaerobic power** | | | |
| **Study** | **Outcome** | **Load indicator** | **Correlation coefficient (*r* value)** |
| Saidi et al. [98] | Mean RSSA time | Weekly sRPE | -0.60 |
|  | Mean RSSA time | Monotony | -0.22 |
|  | Mean RSSA time | Strain | -0.63 |
| Azcárate et al. [99] | ASR | Match sRPEmus | -0.55 |
| Malone et al. [49] | Best time RSA | sRPE | 0.17 |
|  | Best time RSA | iTRIMP | 0.66 |
|  | Best time RSA | luTRIMP | 0.18 |
|  | Best time RSA | eTRIMP | 0.34 |
|  | Best time RSA | bTRIMP | 0.24 |
|  | Best time RSA | gTRIMP | 0.32 |
| Xiong et al. [103] | Mean RSA | sRPE | 0.06 |
|  | Mean RSA | eTRIMP | 0.06 |
|  | Mean RSA | Total distance | 0.20 |
|  | Mean RSA | 14-19VHSR (m) | -0.35 |
|  | Mean RSA | 20VHSR (m) | -0.55 |
|  |  |  |  |
| Notes: ^1^The correlations were performed for specific periods of training. Abbreviations outcome: CMJ (countermovement jump), SJ (squat jump), JS (jump squat), RSSA (repeated shuttle sprint ability), ASR (anaerobic sprint reserve). Abbreviations load indicators: sRPEresp (session rating perceived exertion respiratory), sRPEmus (session rating perceived exertion local-muscular), sRPE_RT_ (session rating perceived exertion resistance training), sRPE_COND_ (session rating perceived exertion conditioning), sRPE_SK_ (session rating of perceived exertion skills), sRPE (session rating perceived exertion), bTRIMP (Bannister’s training impulse), HSD (high speed distance), SD (sprint distance), iTRIMP (individualized training impulse), luTRIMP (Lucia’s training impulse), eTRIMP (Edward’s training impulse), VHSD (very high sprint distance), iHSD (individualized high speed distance), gTRIMP (Stagno individualized training impulse); 13LIRD (low-intensity running distance > 13 km^.^h^-1^); 13-19HIRD (high-intensity running distance 13-19 km^.^h^-1^), 19VHIRD (very high-intensity running distance > 19 km^.^h^-1^). | | | |
