# Supplementary material for: The Correlations Between Training Load Parameters and Physical Performance Adaptations in Team Sports: A Systematic Review and Meta-analysis
Source: Sports Med Open. 2025 Dec 11;11:156. doi: 10.1186/s40798-025-00952-4 (PMC12698923; doi:10.1186/s40798-025-00952-4)
Supplement: Supplementary file 3 — Supplementary Material 3 [file 40798_2025_952_MOESM3_ESM.docx]

| **Supplementary material 4.** Correlations between load and strength outputs. | | | |
| --- | --- | --- | --- |
| **Study** | **Outcome** | **Load indicator** | **Correlation coefficient (*r* value)** |
| **Maximal strength** | | | |
| Clemente et al. [81] | PT knee extension 60º^.^s^-1^ left leg | Weekly sRPE | -0.43 |
|  | PT knee extension 60º^.^s^-1^ left leg | Monotony | -0.08 |
|  | PT knee extension 60º^.^s^-1^ left leg | Strain | -0.65 |
|  | PT knee flexion 60º^.^s^-1^ left leg | Weekly sRPE | -0.01 |
|  | PT knee flexion 60º^.^s^-1^ left leg | Monotony | -0.25 |
|  | PT knee flexion 60º^.^s^-1^ left leg | Strain | -0.26 |
|  | PT knee extension 60º^.^s^-1^ right leg | Weekly sRPE | 0.58 |
|  | PT knee extension 60º^.^s^-1^ right leg | Monotony | 0.60 |
|  | PT knee extension 60º^.^s^-1^ right leg | Strain | 0.04 |
|  | PT knee flexion 60º^.^s^-1^ right leg | Weekly sRPE | 0.14 |
|  | PT knee flexion 60º^.^s^-1^ right leg | Monotony | 0.14 |
|  | PT knee flexion 60º^.^s^-1^ right leg | Strain | 0.01 |
|  | Ratio deficit flexion | Weekly sRPE | -0.87 |
|  | Ratio deficit flexion | Monotony | -0.42 |
|  | Ratio deficit flexion | Strain | 0.01 |
|  | Ratio deficit flexion | Weekly sRPE | 0.13 |
|  | Ratio deficit flexion | Monotony | 0.57 |
|  | Ratio deficit flexion | Strain | -0.20 |
|  | Ratio agonist/antagonist right leg | Weekly sRPE | -0.22 |
|  | Ratio agonist/antagonist right leg | Monotony | -0.12 |
|  | Ratio agonist/antagonist right leg | Strain | -0.25 |
|  | Ratio agonist/antagonist left leg | Weekly sRPE | 0.54 |
|  | Ratio agonist/antagonist left leg | Monotony | 0.22 |
|  | Ratio agonist/antagonist left leg | Strain | 0.28 |
| Clemente et al. [86] | PT quadriceps left leg | Training volume | -0.53 |
|  | PT quadriceps left leg | Total distance | -0.01 |
|  | PT quadriceps left leg | SD | -0.33 |
|  | PT quadriceps left leg | Sum accelerations | 0.67 |
|  | PT quadriceps right leg | Training volume | -0.67 |
|  | PT quadriceps right leg | Total distance | -0.40 |
|  | PT quadriceps right leg | SD | -0.66 |
|  | PT quadriceps right leg | Sum accelerations | 0.19 |
|  | PT hamstring left leg | Training volume | -0.61 |
|  | PT hamstring left leg | Total distance | -0.07 |
|  | PT hamstring left leg | SD | -0.24 |
|  | PT hamstring left leg | Sum accelerations | -0.68 |
|  | PT quadriceps right leg | Training volume | -0.33 |
|  | PT quadriceps right leg | Total distance | -0.18 |
|  | PT quadriceps right leg | SD | -0.33 |
|  | PT quadriceps right leg | Sum accelerations | 0.63 |
|  | Quadriceps/hamstring left leg | Training volume | -0.01 |
|  | Quadriceps/hamstring left leg | Total distance | 0.62 |
|  | Quadriceps/hamstring left leg | SD | -0.88 |
|  | Quadriceps/hamstring left leg | Sum accelerations | 0.11 |
|  | Quadriceps/hamstring right leg | Training volume | 0.31 |
|  | Quadriceps/hamstring right leg | Total distance | 0.74 |
|  | Quadriceps/hamstring right leg | SD | -0.97 |
|  | Quadriceps/hamstring right leg | Sum accelerations | 0.08 |
| Daniels et al. [51] | Bench press | Weekly sRPE | -0.19 |
|  | Back squat | Weekly sRPE | 0.34 |
| **Muscular resistance** | | | |
| **Study** | **Outcome** | **Load indicator** | **Correlation coefficient (*r* value)** |
| Daniels et al. [51] | Pull ups | Weekly sRPE | 0.11 |
| Abbreviation outcomes: PT (peak torque). Abbreviation load indicators: sRPE (session rating perceived exertion), SD (sprint distance). | | | |
