# Supplementary material for: The Correlations Between Training Load Parameters and Physical Performance Adaptations in Team Sports: A Systematic Review and Meta-analysis
Source: Sports Med Open. 2025 Dec 11;11:156. doi: 10.1186/s40798-025-00952-4 (PMC12698923; doi:10.1186/s40798-025-00952-4)
Supplement: Supplementary file 4 — Supplementary Material 4 [file 40798_2025_952_MOESM4_ESM.docx]

| **Supplementary material 5.** Correlations between load and speed outputs. | | | |
| --- | --- | --- | --- |
| **Study** | **Outcome** | **Load indicator** | **Correlation coefficient (*r* value)** |
| **Linear speed** | | | |
| Arcos et al. [95] | 15 m sprint time | total weekly sRPEmus | -0.59 |
| Arcos et al. [92] | 5 m sprint time | sRPEresp | -0.37 |
|  | 5 m sprint time | sRPEmusc | 0.01 |
|  | 5 m sprint time | sumRPEresp | -0.34 |
|  | 5 m sprint time | sumRPEmusc | 0.06 |
|  | 5 m sprint time | Training volume | -0.54 |
|  | 15 m sprint time | sRPEresp | -0.49 |
|  | 15 m sprint time | sRPEmusc | -0.15 |
|  | 15 m sprint time | sumRPEresp | -0.38 |
|  | 15 m sprint time | sumRPEmusc | -0.02 |
|  | 15 m sprint time | Training volume | -0.64 |
| Gil-Rey et al. [60] | 5 m sprint time | sRPEresp | -0.02 |
|  | 5 m sprint time | sRPEmusc | -0.06 |
|  | 5 m sprint time | Training and match volume | 0.23 |
|  | 15 m sprint time | sRPEresp | -0.21 |
|  | 15 m sprint time | sRPEmusc | -0.15 |
| Nakamura et al. [59] | 5 m sprint velocity | Weekly sRPE | -0.18 |
|  | 10 m sprint velocity | Weekly sRPE | -0.26 |
|  | 20 m sprint velocity | Weekly sRPE | -0.27 |
| Arcos et al. [89] | 5 m sprint time | sRPEresp | -0.36 |
|  | 5 m sprint time | sRPEmusc | -0.16 |
|  | 5 m sprint time | sumRPEresp | -0.37 |
|  | 5 m sprint time | sumRPEmusc | -0.15 |
|  | 5 m sprint time | Training volume | -0.05 |
|  | 15 m sprint time | sRPEresp | -0.53 |
|  | 15 m sprint time | sRPEmusc | -0.34 |
|  | 15 m sprint time | sumRPEresp | -0.51 |
|  | 15 m sprint time | sumRPEmusc | -0.24 |
|  | 15 m sprint time | Training volume | -0.23 |
| Dobbin et al. [50] | 10 m sprint time | sRPE_RT_ | -0.52 |
|  | 10 m sprint time | sRPE_COND_ | -0.41 |
|  | 10 m sprint time | sRPE_SK_ | -0.71 |
|  | 10 m sprint time | total sRPE | -0.70 |
|  | 10 m momentum | sRPE_RT_ | 0.12 |
|  | 10 m momentum | sRPE_COND_ | 0.51 |
|  | 10 m momentum | sRPE_SK_ | 0.35 |
|  | 10 m momentum | total sRPE | 0.36 |
|  | 20 m sprint time | sRPE_RT_ | -0.49 |
|  | 20 m sprint time | sRPE_COND_ | -0.65 |
|  | 20 m sprint time | sRPE_SK_ | -0.79 |
|  | 20 m sprint time | total sRPE | -0.77 |
|  | 20 m momentum | sRPE_RT_ | 0.01 |
|  | 20 m momentum | sRPE_COND_ | 0.53 |
|  | 20 m momentum | sRPE_SK_ | 0.27 |
|  | 20 m momentum | total sRPE | 0.29 |
| Fitzpatrick et al. [82] | MSS | sRPE | 0.37 |
|  | MSS | eTRIMP | -0.40 |
|  | MSS | Total distance | 0.46 |
|  | MSS | Acceleration and deceleration distance > 2 m^.^s^-2^ | 0.57 |
|  | MSS | HSD (m) | 0.32 |
|  | MSS | VHSD (m) | 0.25 |
|  | MSS | HSD (min) | 0.34 |
|  | MSS | VHSD (min) | 0.27 |
|  | MSS | Meters above MAS | 0.30 |
|  | MSS | Time above MAS | 0.21 |
|  | MSS | Meters above 30% ASR | -0.09 |
|  | MSS | Time above 30% ASR | -0.15 |
| Malone et al. [49] | 5 m sprint time | sRPE | 0.11 |
|  | 5 m sprint time | iTRIMP | 0.54 |
|  | 5 m sprint time | luTRIMP | 0.20 |
|  | 5 m sprint time | eTRIMP | 0.25 |
|  | 5 m sprint time | bTRIMP | 0.28 |
|  | 5 m sprint time | gTRIMP | 0.22 |
|  | 10 m sprint time | sRPE | 0.13 |
|  | 10 m sprint time | iTRIMP | 0.48 |
|  | 10 m sprint time | luTRIMP | 0.21 |
|  | 10 m sprint time | eTRIMP | 0.22 |
|  | 10 m sprint time | bTRIMP | 0.23 |
|  | 10 m sprint time | gTRIMP | 0.12 |
|  | 20 m sprint time | sRPE | 0.14 |
|  | 20 m sprint time | iTRIMP | 0.66 |
|  | 20 m sprint time | luTRIMP | 0.33 |
|  | 20 m sprint time | eTRIMP | 0.14 |
|  | 20 m sprint time | bTRIMP | 0.35 |
|  | 20 m sprint time | gTRIMP | 0.33 |
| Xiong et al. [103] | 30 m sprint time | sRPE | -0.10 |
|  | 30 m sprint time | eTRIMP | -0.07 |
|  | 30 m sprint time | Total distance | -0.06 |
|  | 30 m sprint time | 14-19VHSR (m) | -0.08 |
|  | 30 m sprint time | 20VHSR (m) | -0.21 |
| Perrotta et al. [105] | 5-m sprint time | Training load Polar | -0.12 |
|  | 5-m sprint time | eTRIMP | -0.12 |
|  | 5-m sprint time | Total distance | -0.17 |
|  | 5-m sprint time | Sprint number | -0.14 |
|  | 5-m sprint time | Acceleration number | 0.10 |
|  | 5-m sprint time | Deceleration number | -0.06 |
|  | 10-m sprint time | Training load Polar | -0.16 |
|  | 10-m sprint time | eTRIMP | -0.15 |
|  | 10-m sprint time | Total distance | -0.22 |
|  | 10-m sprint time | Sprint number | -0.20 |
|  | 10-m sprint time | Acceleration number | 0.16 |
|  | 10-m sprint time | Deceleration number | -0.04 |
| Savolainen et al. [106] | 30-m sprint time | eTRIMP: total duration | 0.11 |
|  | 30-m sprint time | eTRIMP: total distance | 0.08 |
|  | 30-m sprint time | eTRIMP: 13LIRD | 0.06 |
|  | 30-m sprint time | eTRIMP: 13-19HIRD | -0.02 |
|  | 30-m sprint time | eTRIMP: 19VHIRD | -0.09 |
|  | 30-m sprint time | eTRIMP:low acceleration | 0.01 |
|  | 30-m sprint time | eTRIMP: moderate acceleration | 0.02 |
|  | 30-m sprint time | eTRIMP: high aceleration | -0.13 |
|  | 30-m sprint time | HR_MEAN_: total distance | -0.30 |
|  | 30-m sprint time | HR_MEAN_:13LIRD | -0.19 |
|  | 30-m sprint time | HR_MEAN_: 13-19HIRD | -0.18 |
|  | 30-m sprint time | HR_MEAN_: 19VHIRD | -0.40 |
|  | 30-m sprint time | HR_MEAN_: low acceleration | -0.17 |
|  | 30-m sprint time | HR_MEAN_: moderate acceleration | -0.29 |
|  | 30-m sprint time | HR_MEAN_: high aceleration | -0.30 |
|  |  |  |  |
| **Change of direction** | | | |
| **Study** | **Outcome** | **Load indicator** | **Correlation coefficient (*r* value)** |
| Dobbin et al. [50] | COD time | sRPE_RT_ | 0.42 |
|  | COD time | sRPE_COND_ | 0.14 |
|  | COD time | sRPE_SK_ | 0.20 |
|  | COD time | total sRPE | 0.32 |
| Abbreviation outcomes: MSS (maximal sprint speed), COD (change of direction). Abbreviation load indicators: sRPEmus (session rating perceived exertion local-muscular), sRPEresp (session rating perceived exertion respiratory), sRPE (session rating perceived exertion), sRPE_RT_ (session rating perceived exertion resistance training), sRPE_COND_ (session rating perceived exertion conditioning), sRPE_SK_ (session rating of perceived exertion skills), eTRIMP (Edward’s training impulse), HSD (high speed distance), VHSD (very high speed distance), MAS (maximal aerobic speed), iTRIMP (individualized training impulse), luTRIMP (Lucia’s training impulse), bTRIMP (Bannister’s training impulse), gTRIMP (Stagno individualized training impulse); 13-19HIRD (high-intensity running distance 13-19 km^.^h^-1^), 19VHIRD (very high-intensity running distance > 19 km^.^h^-1^); ); 14-19HSRD (high-speed running distance 14-19 km^.^h^-1^), 20VHSRD (very high-intensity running distance covered >20.0 km^.^h^-1^). | | | |
