# Supplementary material for: The Correlations Between Training Load Parameters and Physical Performance Adaptations in Team Sports: A Systematic Review and Meta-analysis
Source: Sports Med Open. 2025 Dec 11;11:156. doi: 10.1186/s40798-025-00952-4 (PMC12698923; doi:10.1186/s40798-025-00952-4)
Supplement: Supplementary file 5 — Supplementary Material 5 [file 40798_2025_952_MOESM5_ESM.docx]

| **Supplementary material 6.** Correlations between load and body composition. | | | | |
| --- | --- | --- | --- | --- |
| **Study** | **Outcome** | **Load indicator** | **Correlation coefficient (*r* value)** |  |
| Granados et al. [55] | Fat free mass | Competition and training volume | 0.62 |  |
| Clemente et al. [86] | Lean mass | Training volume | 0.01 |  |
|  | Lean mass | Total distance | -0.63 |  |
|  | Lean mass | Sprint distance | -0.70 |  |
|  | Lean mass | Sum accelerations | -0.16 |  |
|  | Fat mass | Training volume | 0.26 |  |
|  | Fat mass | Total distance | -0.60 |  |
|  | Fat mass | Sprint distance | -0.77 |  |
|  | Fat mass | Sum accelerations | -0.46 |  |
|  | Body mass | Training volume | 0.24 |  |
|  | Body mass | Total distance | 0.53 |  |
|  | Body mass | Sprint distance | -0.68 |  |
|  | Body mass | Sum accelerations | -0.29 |  |
| Perrotta et al. [105] | Fat mass | eTRIMP | 0.06 |  |
|  | Fat mass | Training load Polar | 0.19 |  |
|  | Fat mass | Total distance | 0.07 |  |
|  | Fat mass | Sprint number | 0.08 |  |
|  | Fat mass | Acceleration number | -0.10 |  |
|  | Fat mass | Deceleration number | 0.04 |  |
|  |  |  |  |  |
